# Anti-Fatigue Peptides from the Enzymatic Hydrolysates of *Cervus elaphus* Blood

**DOI:** 10.3390/molecules26247614

**Published:** 2021-12-15

**Authors:** Jun-Jiang Lv, Yan Liu, Xiao-Yan Zeng, Jia Yu, Yan Li, Xiao-Qin Du, Zhong-Bao Wu, Shi-Lei Hao, Bo-Chu Wang

**Affiliations:** 1 Chongqing Engineering Research Center of Pharmaceutical Sciences, Chongqing Medical and Pharmaceutical College, Chongqing 401331, China; liu.yan908@163.com (Y.L.); zxy1508439@163.com (X.-Y.Z.); 18983230367@163.com (J.Y.); ly20031079@163.com (Y.L.); 2 Bio-Resource Research and Utilization Joint Key Laboratory of Sichuan and Chongqing, Chongqing Institute of Medical Planting Material, Chongqing 408435, China; duxiaoqin1814@sina.com; 3 Key Laboratory of Biorheological Science and Technology, Ministry of Education, College of Bioengineering, Chongqing University, Chongqing 400030, China; shilei_hao@cqu.edu.cn

**Keywords:** deer blood, peptide, anti-fatigue, enzymatic hydrolysis, glutamine

## Abstract

Red deer (*Cervus elaphus*) blood is widely used as a health product. Mixed culture fermentation improves the flavor and bioavailability of deer blood (DB), and both DB and its enzymatic hydrolysates exhibit anti-fatigue activities in vivo. To elucidate the bioactive ingredients, enzymatic hydrolysates were fractioned into different peptide groups using reversed phase resin chromatography, and then evaluated using an exhaustive swimming mice model to assess swimming time and biochemical parameters. The structures of the bioactive peptides were elucidated by high performance liquid chromatography with tandem mass detection. Thirty-one compounds were identified as glutamine or branched-chain amino acids containing short peptides, of which Val-Ala-Asn, Val-Val-Ser-Ala, Leu(Ile)-Leu(Ile)-Val-Thr, Pro-His-Pro-Thr-Thr, Glu-Val-Ala-Phe and Val-Leu(Ile)-Asp-Ala-Phe are new peptides. The fractions containing glutamine or valine short peptides, Ala-Gln, Val-Gln, Val-Val-Ser-Ala, Val-Leu(Ile)-Ser improved exercise endurance by increasing hepatic glycogen (HG) storage. The peptides group containing Leu(Ile)-Leu(Ile), Asp-Gln, Phe- Leu(Ile), Val-Val-Tyr-Pro contributed to decreased muscle lactic acid (MLA)accumulation and to an increase in HG. The anti-fatigue activities of DB hydrolysates were attributed to the synergistic effects of different types of peptides.

## 1. Introduction

Deer farming has an economic value greater than USD 3000 million worldwide [1]. In most countries, the main deer products are antlers and meat, but immature antlers are also important for traditional Chinese medicine in China. As a by-product, deer blood (DB) has negligible economic value in most countries; this is both a waste of nutrients and can have serious environmental impacts [2].

DB is rich in protein [3] (15% in whole blood) and inorganic components, which are beneficial in maintaining health, and it is an important health product for improving fatigue [4] and treating anaemia [5] in China and other Asian countries. However, whole DB can have detrimental effects on the sensory qualities of food, particularly due to its flavor and color. These effects limit the utilization of DB in the food industry. 

An alternative method for using animal blood is to produce peptides by protease hydrolysis. The peptides in animal blood hydrolysate have attracted increasing attention due to their potential bioactivities, such as their antioxidative [6] antimicrobial [7], and angiotensin I converting enzyme (ACE) inhibitory effects [3]. Previous research has mainly focused on the antioxidant peptides generated from porcine plasma [3]. In DB, papain hydrolysate has been reported to have anti-fatigue effects [4]. Moreover, fungal protease (derived from *Aspergillus oryzae*) hydrolyzed DB peptides exhibit antioxidative activities [6]. Probiotics-fermented DB ameliorated intense exercise-induced fatigue [8]. Liu et al. [9]. investigated the degree of DB hydrolysis in six different enzymes. However, no study has investigated the anti-fatigue effects of DB hydrolysate in mixed enzymes, nor have any studies investigated the relationships between peptide structure and anti-fatigue effects.

In this study, mixed enzymes (papain, neutral protease, alcalase, and flavourzyme) were used to hydrolyze DB. The peptides were fractioned and their anti-fatigue bioactivities were evaluated. The structures of the active peptides were then elucidated by high performance liquid chromatography with tandem mass detection (HPLC-MS/MS), and the structure-activity relationships were discussed.

## 2. Results and Discussion

### 2.1. Enzymatic Hydrolysis of DB and Chromatography Fraction of DB Hydrolysates

**Figure 1:**
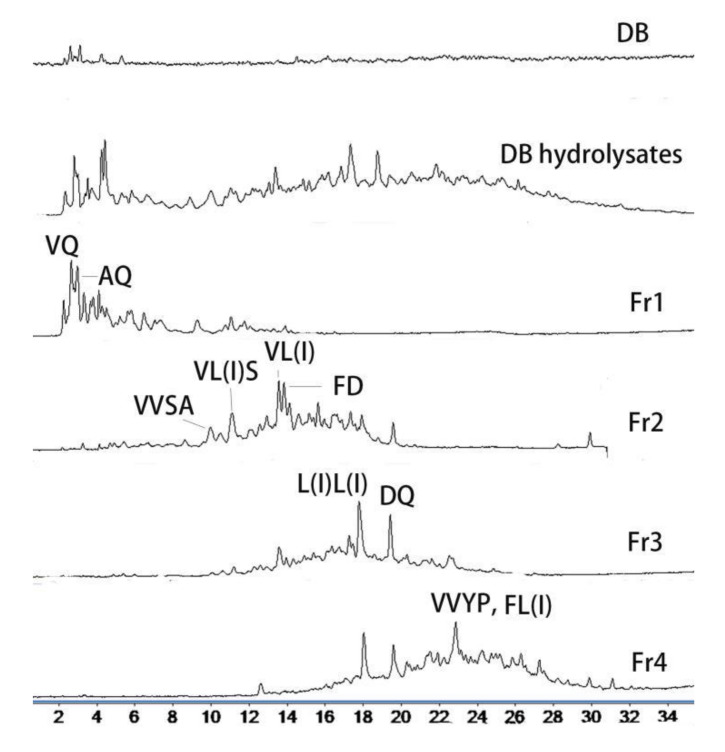
HPLC-MS chromatogram of deer blood (DB) hydrolysates and fractions.

To improve its undesirable flavor, as well the bio-availability of deer blood, mixed enzymes were used to hydrolyze the protein into peptides. As seen in Figure 1, the enzymatic hydrolysates of DB contained numerous small molecules in comparison to the flat HPLC chromatogram of DB. The retention time of the peptides was mainly at 3–7 min and 15–30 min. The molecular mass of the peptides was mainly distributed between 200 and 600 Da, as determined by HPLC-ESI-MS.

To investigate the anti-fatigue activities of the different peptides, the DB hydrolysates were fractioned via resin chromatography (NM 200). The resin NM 200 was a Poly(styrene-co-divinylbenzene) reversed phase material, and the surface groups were benzyls. Thus, the peptides mixture could be fractioned mainly based on the Van der Waals interaction caused by different skeleton structures. The HPLC-MS chromatogram (Figure 1) demonstrated that Fr1, Fr2, and Fr4 were comprised of different ingredients, with retention times of 3–7, 10–20, and 18–28 min, respectively. Fr3 was a mixture of Fr2 and Fr4. Thus, the DB hydrolysates were divided into three fractions of peptides with different chemical properties. The weight of Fr1 was 7.1 g, which was 47% of the total weight of enzymatic hydrolysates. Fr2, 3 and 4 weighed 1.9 g, 2.8 g, and 3.3 g, respectively. It can be noted that nearly half the peptides were distributed in Fr1.

### 2.2. Anti-Fatigue Evaluation

Physiological fatigue depends on the energy metabolism of muscular activity [10,11]. The evaluation of fatigue mainly focuses on using endurance experiments and biochemical determination. Exercise endurance time is commonly used to evaluate endurance in animals; it is considered to be the most objective and easy-to-operate model for evaluating anti-fatigue effects [12,13]. Energy metabolism is usually evaluated using biochemical assays. BUN (Blood urea nitrogen), HG (Hepatic glycogen) and MLA (Muscle lactic acid) are sensitive to physiological fatigue and are widely used for anti-fatigue drug and food screening [14,15].

#### 2.2.1. Effects on Body Weight and Swimming Time

Over the 10 days of the experiment, the body weights of the mice in all groups increased, along with their feeding time; however, there were no significant differences between the groups (*p* < 0.05). This result suggested that the DB and DB peptides had no effects on the mice’s body weights (Figure S1).

**Figure 2:**
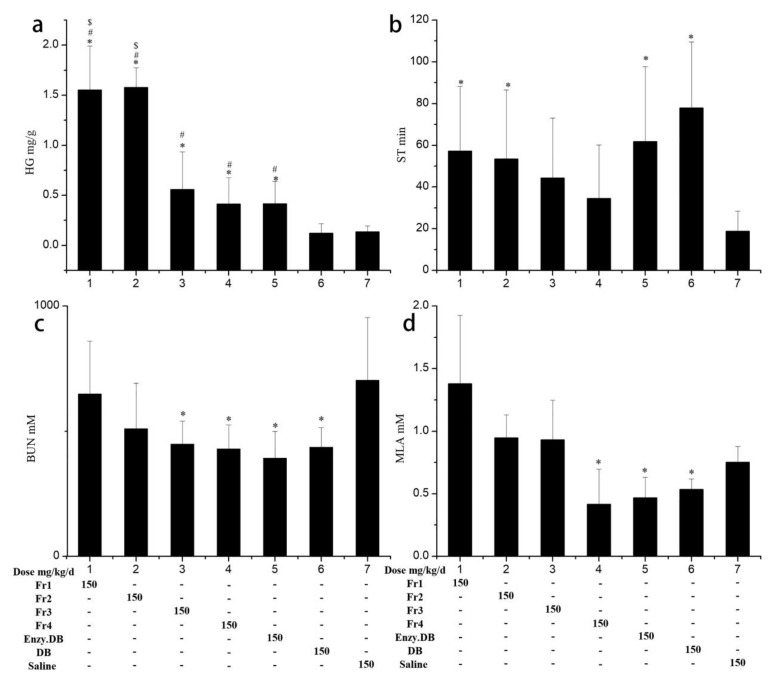
Hepatic glycogen (HG) (**a**), swimming to exhaustion time (ST) (**b**), blood urea nitrogen (BUN) (**c**), and muscle lactic acid (MLA) (**d**), of mice loaded with a lead sheet (5% of their body weight) swimming in 25 °C water (swimming time for BUN, MLA and HG was 18 min) for each of the 7 groups of mice (see text for treatment explanation). Data are expressed as means ± SD (n = 8). * *p* < 0.05, compared with the saline group. # *p* < 0.05 compared with the DB group. $ *p* < 0.05 compared with the deer blood (DB) hydrolysates group.

An increase in exercise endurance is a direct reflection of anti-fatigue effect. We constructed an exhaustive swimming exercise mice model to evaluate exercise endurance. In the pre-experiments, we noted that water temperature and loaded weight could significantly influence the experimental results. When the loaded weight was 7.5% of the mouse’s body weight, all the mice became exhausted within 2 min, and the swimming time was too short to demonstrate any differences between groups. When the loaded weight was less than 3% of the mouse’s body weight, it was too light, such that the mice were able to float on the water surface without any exercise. Thus, a loaded weight 5% of the mouse’s body weight avoided floating while also allowing for the maintenance of a proper swimming time. With 5% of the body weight loaded to the tail ends, the mice’s swimming was sensitive to the temperature of the water. For instance, mice were able to swim for as long as 40 min in 29 °C water, but only 20 min in 25 °C water. Thus, accounting for the lower deviation with shorter experimental time for the exhaustive swimming time test, a loaded weight of 5% of the body weight of each mouse and a water temperature of 25 °C were selected. The results (Figure 2b) showed that DB, DB hydrolysates, Fr1, and Fr2 significantly improved the swimming to exhaustion time (*p* < 0.05) by 315%, 271%, 204%, and 185%, respectively, compared with the saline group. This suggests that the DB enzymatic hydrolysates had similar effects to DB in terms of improving swimming to exhaustion times. For the hydrolyzed peptides, Fr1 and Fr2 led to increase swimming times, suggesting that the anti-fatigue effects of DB enzymatic hydrolysates were attributed to different kinds of peptides.

#### 2.2.2. Effects on BUN, HG, and MLA

BUN is the metabolite of proteins and acids, which is generated when sugar and fat are not able to provide enough energy for the body under high-intensity exercise. BUN is inversely sensitive to physical fatigue. In mice, BUN is produced after long periods of free swimming [16,17]; however, we found that when the mice with no weight-load swam in 30 °C water, they preferred to float rather than swim, even though we continually disturbed the water’s surface. Using this method, it would be difficult to force the mice to completely exercise, leading to large deviations in the experimental data. Thus, we optimized the procedure for the biochemical assay for anti-fatigue evaluations. According to the pre-experimental results, we noted that when the mice swam for a time interval (18 min) close to the swimming to exhaustion time (19 min for the saline group) in 25 °C water with a 5% body weight load, there were significant differences in all three parameters (i.e., BUN, HG, and MLA) between the groups. Using this method, we collected samples for all three biochemical parameters in one batch of mice within 20 min; this differed from previously reported procedures, in which one group of mice swam for 90 min for BUN and HG detection, and another group of mice swam with a load for 10 min for MLA detection [16].

Since BUN is inversely related to anti-fatigue effects, a lower BUN level represents better exercise tolerance. The results in Figure 2c indicated that the DB and DB hydrolysates groups displayed the lowest levels of BUN, and both were significantly lower than those of the saline group (*p* < 0.05). Fr3 and Fr4 also had lower BUN levels than those of the saline group (*p* < 0.05). Together, this suggests that both DB and DB hydrolysates can improve exercise tolerance and that the peptides in the Fr3 and Fr4 fractions were the active ingredients contributing to decreased BUN in the DB hydrolysates group.

Glucose is an important energy source, and exercise fatigue is always accompanied by muscle glycogen exhaustion. During high-intensity exercise, to keep a constant blood glucose concentration, HG is consumed, and its store decreases. HG is sensitive to physical fatigue, and it is widely used as a biochemical parameter in the evaluation of anti-fatigue foods and drugs [18]. The results are shown in Figure 2a. DB had no effect on HG storage. Mice in the DB hydrolysates group exhibited 307% higher HG storage compared with those of the saline group (*p* < 0.05). Mice from all the DB hydrolysate fraction groups had increased HG storage, particularly those in the Fr1 and Fr2 groups, with HG levels of 1.56 ± 0.44 and 1.58 ± 0.20 mg/g, 12 times higher than those of the DB and saline groups. Results suggest that the peptides in the DB hydrolysates that can increase HG storage are mainly enriched in Fr1 and Fr2.

Lactic acid is generated through the glycolysis pathway during severe exercise. The accumulation of lactic acid leads to physical fatigue and depends on the relative speed of lactic acid generation and elimination. Thus, either decreasing the generation or increasing the elimination of lactic acid can relieve the degree of fatigue [19]. The MLA results (Figure 2d) illustrate that both DB and DB hydrolysates can decrease lactic acid (*p* < 0.05) by 44% and 45%, respectively, compared with the levels shown in the saline group. For the four fractions, only the peptides in Fr4 decreased lactic acid accumulation, indicating that the active peptides that eliminate MLA were found in fraction Fr4.

The anti-fatigue evaluation results are summarized in Table 1. In comparison with the saline group, both the DB and DB hydrolysates groups exhibited increased exercise times and decreased BUN and MLA levels. Additionally, the DB hydrolysates group had increased HG storage. Thus, these results suggest that DB hydrolysates have superior anti-fatigue activities compared with DB powder. The four DB hydrolyzed peptide fractions had different anti-fatigue effects. Fr1 and Fr2 improved exercise induration and increased HG storage but had no effects on BUN and MLA levels, whereas Fr3 and Fr4 increased HG storage and decreased MLA levels but had no effects on exercise duration. Thus, we conclude that the anti-fatigue effects of the DB enzymatic peptides are attributed to several peptides with different structures rather than to a single peptide; in other words, different peptides derived from DB can have synergistic effects. Thus, to explain the structure–effects relationships, the structures of the peptides in Fr1, Fr2, and Fr4 were elucidated by HPLC-MS/MS.

### 2.3. Identification of Bioactive Peptides

**Figure 3:**
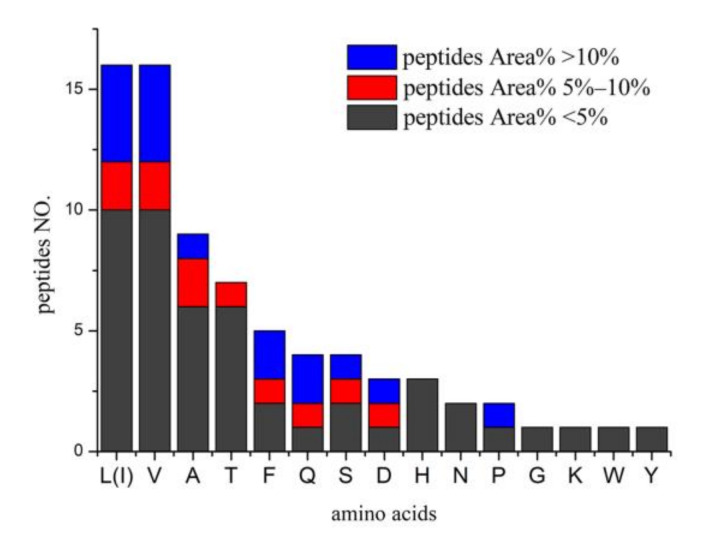
Amino acids compositions of peptides from DB hydrolysates. The vertical axis represent the number of peptides containing the amino acids (horizontal axis), L(I) leucine(isoleucine), V (valine), A (alanine), T (threonine), F (pheylalanine), Q (glutmine), S (serine), D (aspartic acie), H (histidine), N (asparagine), P (proline), G (glycine), K (lysine), W (tryptophan), and Y (tyrosine).

The MS spectra of Fr1, Fr2, and Fr4 are shown in Figure 1; these three fractions were comprised of different types of peptides. As identified by HPLC-MS/MS analysis, Fr1, Fr2, and Fr4 had 31 peptides (Table 2). VAN, VVSA, L(I)L(I)VT, PHPTT, EVAF and VL(I)DAF are new peptides. These peptides were formed by 16 amino acids (Figure 3), and 25 peptides contained branched-chain amino acids (BCAAs). It can be noted from Figure 3 that there were 16 leucine/isoleucine and valine-containing peptides, respectively; six of each group were above 5% in content. Of the eleven main peptides (Area% > 5%), eight contained BCAAs. This suggests that the DB hydrolysates derived from mixed enzymatic hydrolysis are mainly comprised of BCAA-containing peptides. Besides, nine peptides contained Ala and four contained Gln (Figure 3). BCAAs valine, isoleucine, and valine are known to have various anti-fatigue effects, and many studies have evaluated the effects of BCAAs [20].

**Figure 4:**
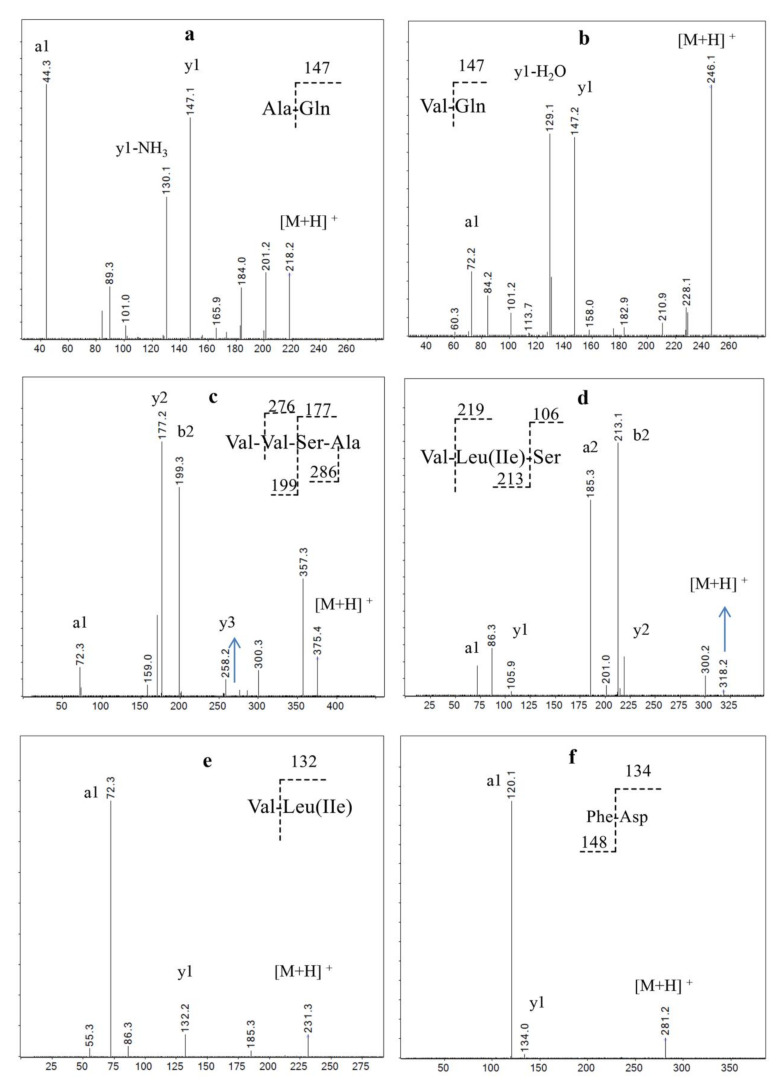
MS/MS spectra analysis of the main peptides Fr1and Fr2, (**a**) Ala-Gln, (**b**) Val-Gln, (**c**) Val-Val-Ser-Ala, (**d**) Val-Leu(Ile)-Ser, (**e**) Val-Leu(Ile), (**f**) Phe-Asp.

Fr1 is the main fraction of DB hydrolysate (47% of the total weight), in which AQ and VQ are major components (Figure 1 and Figure 4; Table 2). Glutamine and alanine are well-known glycogenic amino acids in animals and humans [21], and glutamine is also a direct stimulator of glycogen synthesis via the activation of glycogen synthetase [22,23]. Dipeptide AQ has been widely used as a commercial supplement to increase glutamine availability. It is reported that dipeptides and tripeptides are absorbed across the intestinal epithelium in their intact form via a more efficient mechanism than that of free amino acids [24,25]. Both glutamine and alanyl-glutamine significantly increase glycogen storage in exhaustive swimming rats but have no effects on plasma ammonia [26] or lactic acid accumulation [27]; this agrees with the result that Fr1 significantly increased HG but had no effects on MLA and BUN levels. Thus, the strong effects of Fr1 on improving exercise endurance are likely to be attributed to AQ and VQ through increased HG storage.

Fr2 is mainly comprised of VVSA, VL(I)S, VL(I), and FD (Figure 1 and Figure 4; Table 2). Three of the four major peptides are valine-containing di-, tri-, and tetra-peptides. VL is a minor dipeptide derived from milk whey protein enzymatic hydrolysates; it can inhibit NF-kB expression, suggesting an anti-inflammatory effect on inflammation caused by acute exercise, reduced glucagon-like peptide-1(GLP-1), and glucose-dependent insulinotropic polypeptide (GIP) with no effects on liver glycogen [28]. However, studies of valine using swimming rat models have shown that it is effective for maintaining HG but has no effect on lactic acid [29]. FD was reported to be effective against SHP2 enzymatic activity, which is found to be overexpressed in breast cancer cell lines [30]. There are no reports on the anti-fatigue effects of VVSA, VL(I)S, FD, L(I)VT, and other minor peptides in Fr2 (Table 2). Our results suggested that these valine-containing short peptides can improve exercise endurance by maintaining glycogen storage.

**Figure 5:**
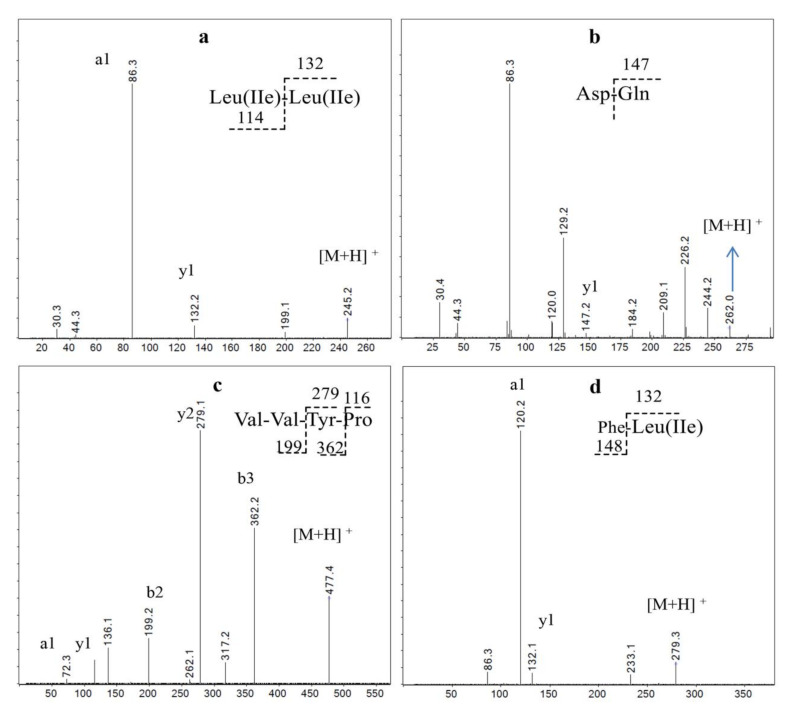
MS/MS spectra analysis of the main peptides in Fr4, (**a**) Leu(Ile)-Leu(Ile), (**b**) Asp-Gln, (**c**) Val-Val-Tyr-Pro, (**d**) Phe-Lue(Ile).

The major components of Fr4 are L(I)L(I), DQ, FL, and VVYP; the L(I)L(I) content was 11.6% (Figure 1 and Figure 5; Table 2). Leucine and isoleucine promote glucose uptake and utilization [31], which can deplete blood glucose. Tsuda et al. [29]. reported that the administration of leucine significantly decreased blood glucose levels, which may worsen physical condition and augment physical fatigue. It was reported by Morifuji [32] that in Ile-Leu, the main BCAA-containing dipeptides in whey-stimulated insulin, independent glucose uptake in both L6 myotubes and isolated epitrochlearis muscles, possibly by PI3-kinase and aPKC pathways, resulted in increased skeletal glycogen contents. Consistent with this view, Moura et.al. [28] reported that Ile-Leu was involved in an increase in liver and muscle glycogen, provided anti-stress effect and attenuated exercise-induced immunosuppression. Using the acute treadmill exercise training rats model, Morato [33] found that Leu-Ile and L in whey can increase the translocation of the major glucose transporter, GLUT-4, and the entrance of glucose into skeletal muscle; but had no effects on liver and muscle glycogen. Our results showed that while both Fr3 and Fr4 with the major components L(I)L(I) displayed HG increase effects compared to the saline group (*p* < 0.05), they were lower than those of Fr1 and Fr2 (*p* < 0.05, Figure 2). The results suggest that L(I)L(I) has an HG increase effect, but it is lower than that of the glutamine- or valine-containing peptides in Fr1 and Fr2. This may explain the lower exercise endurance in Fr3 and Fr4 groups compared to the Fr1 and Fr2 groups. DQ, as a glutamine-containing dipeptide, was another main ingredient in Fr3 and Fr4, but there is no report on its bioactivities. Thus, L(I)L(I) and DQ may contribute to BUN decrease and HG increase effects of Fr3 and Fr4, since they are the major peptides in these two fractions. Among the four fractions, only Fr4 decreased lactic acid. Compared with Fr3, VVYP and FL(I) were the characteristic major components (15.2%), such that VVYP and FL(I) may be the bioactive ingredient that inhibits increases in MLA. FL was found to have anxiolytic-like activity [34]; VVYP, derived from globin hydrolysates by acidic protease, was reported to have hypotriglyceridemic effects by inhibiting fat absorption from the digestive tract and enhancing activity of hepaticlipase [35]. However, there is no anti-fatigue research on FL(I), YYYP, DQ, and other minor peptides in Fr4.

## 3. Materials and Methods

### 3.1. Chemicals and Reagents

The organic solvents that were used for isolation were analytical grade (Chendu Kelong Co., Chengdu, China). The mobile phase of HPLC and HPLC-MS used chromatography grade reagents (Merck Co., Darmstadt, Germany). Water was treated in a Milli-Q water purification system (Millipore, Bedford, MA, USA). Column chromatography was performed with NM-200 (Poly(styrene-co-divinylbenzene resin, 200–500 μm, Suzhou Nanomicro Technology Co., Ltd., Suzhou, China). A 250 × 4.6 mm, i.d. 5 µm, COSMOSIL PBr column (NACALAI TESQUE, Inc., Kyoto, Japan) was used for HPLC and HPLC-MS analysis. Mixed protease (a mixture of papain, neutral protease, alcalase, and Flavourzyme used for animal proteins; 1500 units/g) was purchased from China PangBo Biological Engineering (Guangxi, China). The assay kits used to determine blood urea nitrogen (BUN), hepatic glycogen (HG), and muscle lactic acid (MLA) were purchased from Nanjing Jiancheng Biotechnology Institute (Nanjing, China).

### 3.2. Materials

Whole DB was obtained from Jilin Jiluyuan Bio-Tech (Jilin, China) as a freeze-dried powder. Red deer (*Cervus elaphus*) were 16–18 months of age at the time of collection.

### 3.3. Enzymatic Hydrolysis of DB

The enzymatic hydrolysis procedure was conducted according to the previously reported method with a slight modification [36]. A combination of 15 g of freeze-dried, powdered DB, 100 mL of distilled water, and 80 mg of mixed protease was added to a beaker. The reactants were stirred in a water bath (50 °C) for 6 h. Then, the enzymes were inactivated at 90 °C for 20 min. The enzymatic hydrolysates were cooled to 20 °C, and 100 mL 60% ethanol was added and allowed to stand at room temperature for 24 h. Finally, filtration was performed to remove all insoluble components, and the final pH of the hydrolysate was 7.4. The enzymatic hydrolysates were stored at 4 °C.

### 3.4. Fractioning of Enzymatic Hydrolysates

The enzymatic hydrolysates were fractioned on resin column NM 200 (i.d. 7.0 × 35 cm), then eluted with CH_3_OH/H_2_O (0%, 10%, 30%, 50%, 70%) at a volume of 1.5 L for each gradient to produce four fractions: Fr1 7.1 g, Fr2 1.9 g, Fr3 2.8 g, and Fr4 3.3 g.

### 3.5. HPLC and HPLC-MS Detection

HPLC and HPLC-MS analysis were performed on an Agilent 1290 chromatograph equipped with a DAD detector and a 6460 Triple Quad MS detector (Agilent Technologies, Santa Clara, CA, United States). A PBr column (250 × 4.6 mm, i.d. 5 µm, COSMOSIL, NACALAI TESQUE, Inc., Kyoto, Japan) was used for peptide analysis.

The column was thermostated at 25 °C, with a mobile phase flow rate of 1.0 mL/min. Gradient elution mode was performed using a double solvent system composed of A: 80% ACN/0.05% TFA and B: 0.05% TFA. The gradient was 0–5 min A/B (a:b 5:95), 5–40 min A/B (a:b 5:95–60:40), 40–53 min A/B (a:b 60:40–100:0), 53–53.1 min A/B (a:b 100:0–5:95), and 53.1–60 min A/B (a:b 5:95). Absorbance was recorded at 220 nm. For HPLC-MS and HPLC-MS/MS analysis, the gradient was 0–5 min A/B (a:b 5:95), 5–40 min A/B (a:b 5:95–60:40), 40.1 min A/B (a:b 5:95), 47 min A/B (a:b 5:95).

HPLC-MS/MS analysis was performed in positive ionization modes using full-scan mode and mass range set at *m/z* 50–1500. The HPLC effluent was introduced into an electrospray source with the following conditions: gas temperature: 350 °C; gas flow: 11 L/min; capillary voltage: 4 kV; cone voltage: 80 V; collision energy: 5–30 eV. All operations, and the acquisition and analysis of data, were controlled by Agilent MassHunter Workstation software, Version B.08.02, 2015 (Agilent Technologies, Santa Clara, CA, United States).

In terms of sample preparation, parts of the extract and fraction solutions were evaporated to dry in a vacuum. Then, 25 mg of the residue was dissolved in methanol and diluted to a constant volume of 25 mL, which was microfiltered into a HPLC vial.

### 3.6. Experimental Animals and Anti-Fatigue Studies

Male Kunming mice (22–25 g) were purchased from the Laboratory Animal Center of Third Military Medical University (Chongqing, China).

A total of 112 mice were randomly divided into 7 groups (n = 16 per group; each group was randomly divided into two subgroups, with 8 mice used for the exhaustive swimming test and 8 mice used for biochemical analysis). From days 1–10, groups 1–7 were orally treated with Fr1 (group 1), Fr2 (group 2), Fr3 (group 3), Fr4 (group 4), DB enzymatic hydrolysates (group 5), DB powder (group 6), or saline (group 7) at a dose of 150 mg/kg/day, respectively (each day at 9:30–10:00). During the experimental process, the mice were provided with a standard laboratory diet and water, and the body weight was recorded on day 1 and 10, respectively.

### 3.7. Exhaustive Swimming Test

From each group, 8 mice were used for the exhaustive swimming test 30 min after the last oral administration. A lead sheet (5% of their body weight) was tied to the tail root of each mouse, and the mouse was placed in a swimming tank (50 × 50 × 40 cm) with water kept at 25 °C. Exhaustion time was recorded as the time when the mice failed to rise to the water’s surface within 10 s.

### 3.8. Biochemical Analysis

The other 8 mice from each group were made to swim with a lead sheet tied to their tail roots (5% of their body weight) 30 min after the last oral administration in the same swimming tank (50 × 50 × 40 cm, water at 25 °C). After 18 min, all mice were removed from the water. Blood, liver, and hind leg muscle samples were immediately collected to determine BUN, HG, and MLA levels using testing kits, according to the protocols provided by the manufacturers.

## 4. Conclusions

DB hydrolysate is a rich source of anti-fatigue peptides. The fractions of glutamine- and BCAA-containing peptides can significantly improve the exercise endurance of mice. Compared with DB, DB hydrolysates can significantly increase glycogen storage, which suggests that they have more potent anti-fatigue qualities. The DB hydrolysate fractions and HPLC-MS/MS analysis confirmed that fractions containing VQ and AQ, or VVSA, VL(I)S, VL(I), L(I)VT and FD, can improve glycogen storage. Additionally, fractions abundant in L(I)L(I) and DQ are linked to a reduction in BUN. Fractions containing FL and VVYP may be effective in decreasing MLA. The anti-fatigue activities of DB hydrolysates were attributed to the synergistic effects of these peptides. Thus, DB hydrolysates can be a good source for the development of novel functional food.

## Figures and Tables

**Table 1:** Summary of the anti-fatigue effects of DB and its peptides.

Groups	ST ^a^	BUN ^b^	HG ^c^	MLA ^d^
Fr1	+ ^f^	−	+	−
Fr2	+	−	+	−
Fr3	− ^g^	+	+	−
Fr4	−	+	+	+
DB hydrolysates	+	+	+	+
DB ^e^	+	+	−	+

**^a^** Swimming time; **^b^** Blood urea nitrogen; **^c^** Hepatic glycogen; **^d^** Muscle lactic acid; **^e^** Deer blood; **^f^** *p* < 0.05 compared with saline group; **^g^** not significant.

**Table 2:** Peptide structures.

No.	Fr1			Fr2			Fr3			Fr4		
	RT ^a^	Peptides	Area% ^b^	RT	Peptides	Area%	RT	Peptides	Area%	RT	Peptides	Area%
1	2.7	VQ	19.0	9.9	VVSA	9.1	11.1	V L(I)S	2.7	18.0	L(I)L(I)	11.6
2	2.9	AQ	10.6	11.1	V L(I)S	11.1	13.5	V L(I)	9.1	19.5	DQ	7.4
3	3.3	L(I)H VN GVH	5.0	12.0	VNQ(K)	3.0	14.1	L(I)VT	3.0	20.9	ARVT	1.2
4	3.7	VT	2.9	13.5	V L(I)	11.6	14.6	L(I)VE	2.4	21.4	AYPTT	4.3
5	3.8	VAN	4.4	13.8	FD	10.1	16.5	L(I)SAL(I)	1.7	22.8	VVYP FL	15.2
6	4.2	TVA L(I)Q	3.2	14.1	L(I)VT	5.8	17.3	AF	5.7	24.2	WT	4.3
7	4.4	L(I)	3.5	14.6	L(I)VE	3.6	18.0	L(I)L(I)	26.2	25.0	EVAF	0.9
8	4.9	L(I)T	2.3	16.5	L(I)SAL(I)	4.8	19.5	DQ	13.2	25.8	MTH	2.6
9	8.9	AL(I)	4.4	17.3	AF	5.0				27.2	VNDAF	3.3
10	11.5	L(I)SE	2.8	18.0	L(I)L(I)	3.8						

**^a^** retention time; **^b^** peptides contents with area normalization method.

## Data Availability

Data is contained within the article and the Supplementary Materials.

## Supplementary Materials

The following are available online. Figure S1: Body weight of experimental mice, MS/MS spectra of identified peptides.

## Sample Availability

Samples of the compounds are not available from the authors.

## Abbreviations

BUN	Blood urea nitrogen
DB	Deer blood
HG	Hepatic glycogen
MLA	Muscle lactic acid
