# Supplementary material for: Anti-Fatigue Peptides from the Enzymatic Hydrolysates of Cervus elaphus Blood
Source: Molecules. 2021 Dec 15;26(24):7614. doi: 10.3390/molecules26247614 (PMC8708016; doi:10.3390/molecules26247614)

Supporting Information Available

Anti-fatigue peptides from the enzymatic hydrolysates of *Cervus elaphus* blood

Jun-Jiang Lv [1]*, Yan Liu [1], Xiao-Yan Zeng [1], Jia Yu [1], Yan Li [1], Xiao-Qin Du[2], Zhong-Bao Wu [2]*, Shi-Lei Hao [3], Bo-Chu Wang [3]*

[1]*Chongqing Engineering Research Center of Pharmaceutical Sciences, Chongqing Medical and Pharmaceutical College, Chongqing 401331, China.*

[2]*Bio-resource Research and Utilization Joint Key Laboratory of Sichuan and Chongqing, ChongqingInstitut e of Medical Planting Material, Chongqing 408435, China*

[3]*Key Laboratory of Biorheological Science and Technology, Ministry of Education, College of Bioengineering, Chongqing University, Chongqing 400030, China.*

* Corresponding author. Fax and telephone: (+) 86-023-61969068.

E-mail address: ljjcqu@126.com (J.J. Lv), wangbc2000@126.com (B.C. Wang), wuzhongbao7788@sina.com (Z.B. Wu)

Table of contents

1.	S1 Body weight of experimental mice	3
2.	S2 MS/MS spectrum of VQ	4
3.	S3 MS/MS spectrum of AQ	5
4.	S4 MS/MS spectrum of VVSA	6
5.	S5 MS/MS spectrum of V L(I)S	6
6.	S6 MS/MS spectrum of V L(I)	7
7.	S7 MS/MS spectrum of FD	8
8.	S8 MS/MS spectrum of L(I)L(I)	10
9.	S9 MS/MS spectrum of DQ	10
10.	S10 MS/MS spectrum of VVYP	11
11.	S11 MS/MS spectrum of FL(I)	12
12.	S12 MS/MS spectrum of VAN	13
13.	S13 MS/MS spectrum of L(I)L(I)VT	14
14.	S14 MS/MS spectrum of PHPTT	15
15.	S15 MS/MS spectrum of EVAF	16
16.	S16 MS/MS spectrum of VL(I)DAF	17
17.	S17 MS/MS spectrum of L(I)H	18
18.	S18 MS/MS spectrum of VN	19
19.	S19 MS/MS spectrum of VGH	20
20.	S20 MS/MS spectrum of VT	21
21.	S21 MS/MS spectrum of TVA	22
22.	S22 MS/MS spectrum of L(I)Q	23
23.	S23 MS/MS spectrum of L(I)	24
24.	S24 MS/MS spectrum of L(I)T	25
25.	S25 MS/MS spectrum of AL(I)	26
26.	S26 MS/MS spectrum of L(I)SE	27
27.	S27 MS/MS spectrum of VL(I)K	28
28.	S28MS/MS spectrum of L(I)VT	29
29.	S29 MS/MS spectrum of L(I)VE	30
30.	S30MS/MS spectrum of L(I)SAL(I)	31
31.	S31 MS/MS spectrum of AF	32
32.	S32 MS/MS spectrum of WT	33

1. S1 Body weight of experimental mice

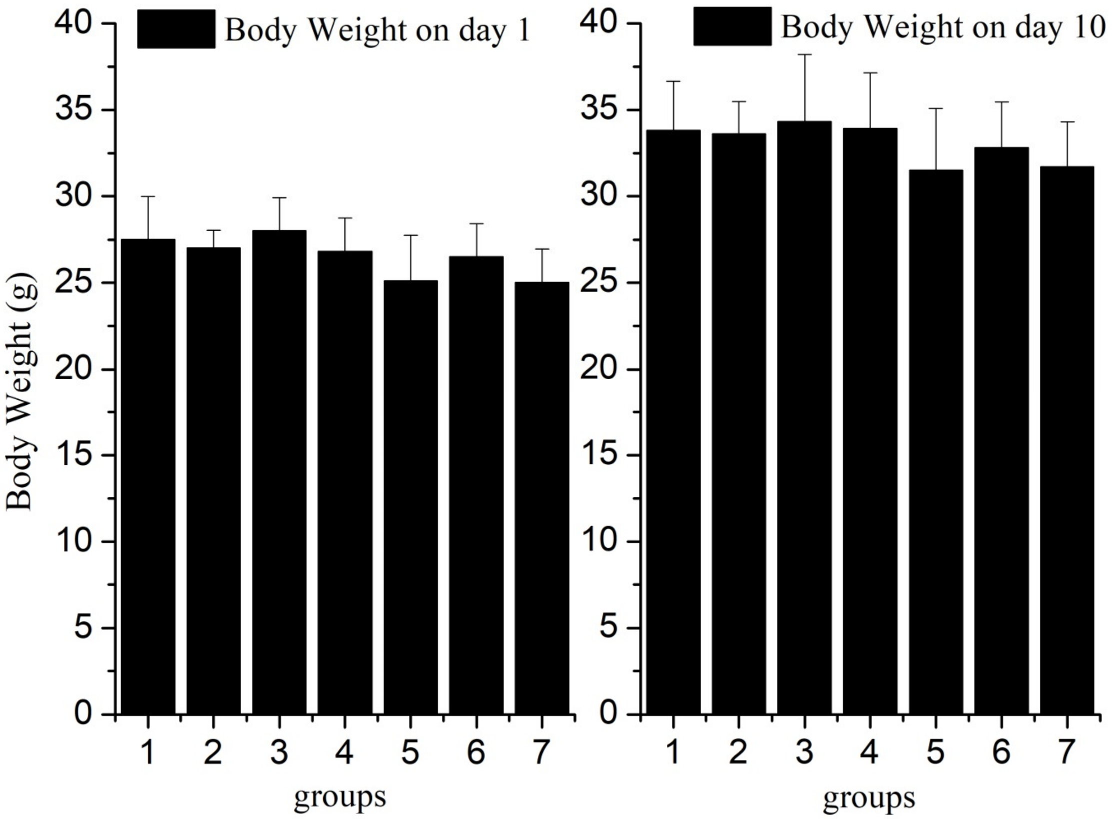

2. S2 MS/MS spectrum of VQ

Sample Name	LX-nm-1-MSMS	Position	P1-A1	Instrument Name	Instrument 1
User Name	YU JIA	Inj Vol	10	InjPosition	
Sample Type	Sample	IRM Calibration Status	Not Applicable	Data Filename	LX-nm-1MSMS.d
ACQ Method	LX-NM-1-MSMS.m	Comment		Acquired Time	6/9/2020 4:57:54 PM (UTC+08:00)

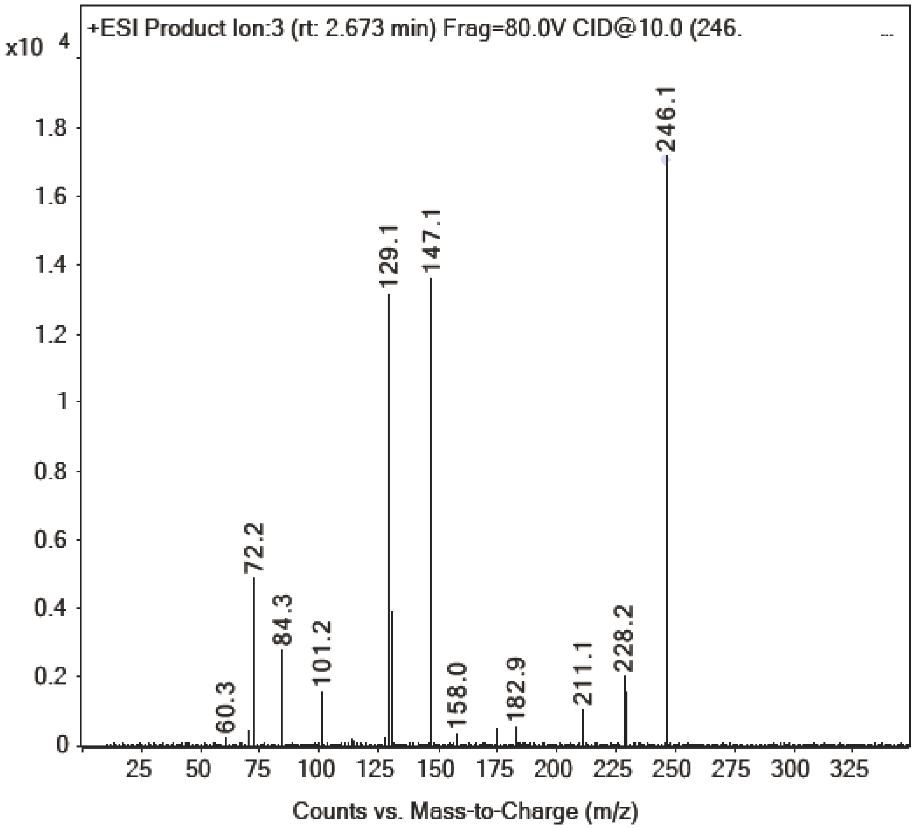

3. S3 MS/MS spectrum of AQ

Sample Name	LX-nm-1-MSMS	Position	P1-A1	Instrument Name	Instrument 1
User Name	YU JIA	Inj Vol	10	InjPosition	
Sample Type	Sample	IRM Calibration Status	Not Applicable	Data Filename	LX-nm-1MSMS.d
ACQ Method	LX-NM-1-MSMS.m	Comment		Acquired Time	6/9/2020 4:57:54 PM (UTC+08:00)

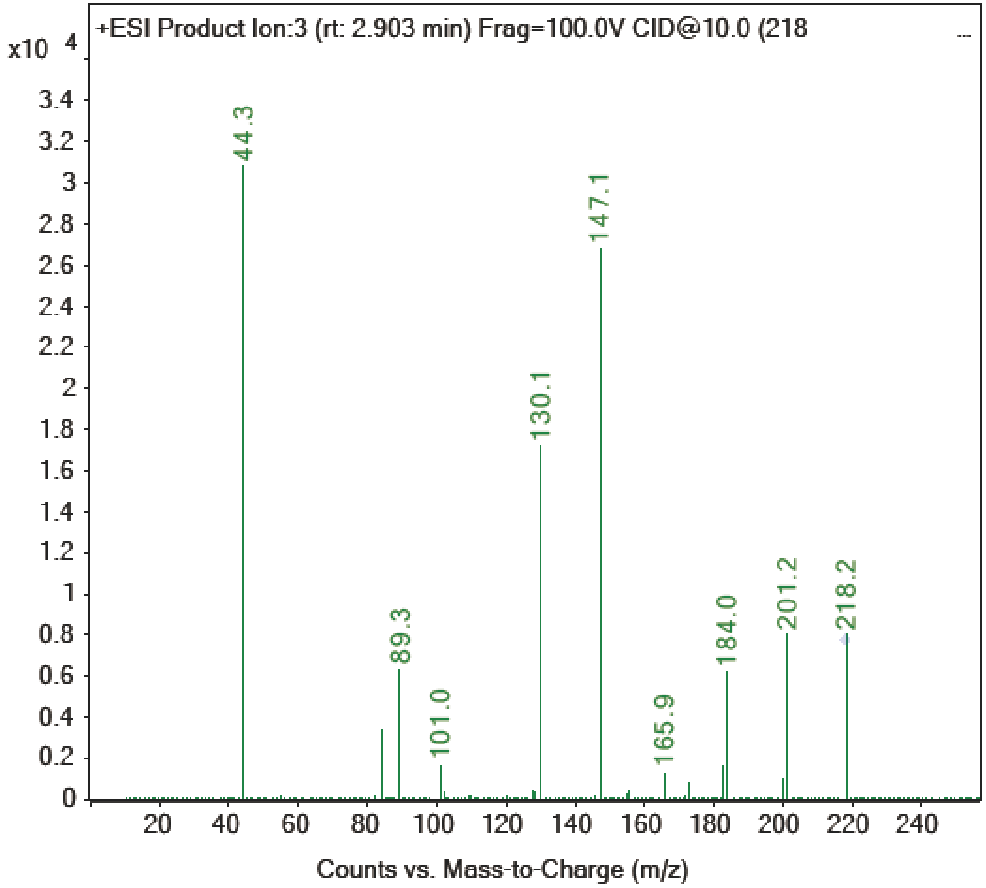

4. S4 MS/MS spectrum of VVSA

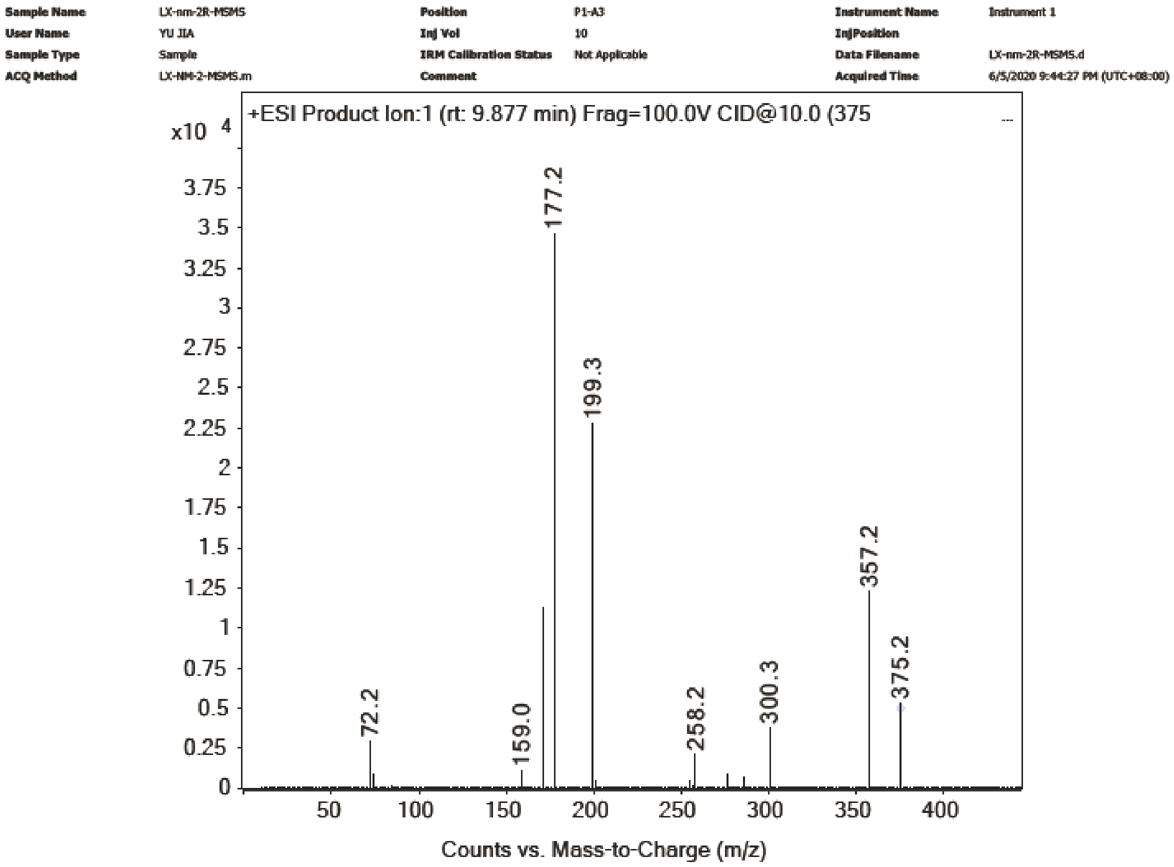

5. S5 MS/MS spectrum of V L(I)S

Sample Name	LX-nm-2R-MSMS	Position	P1-A3	Instrument Name	Instrument 1
User Name	YU JIA	Inj Vol	10	InjPosition	
Sample Type	Sample	IRM Calibration Status	Not Applicable	Data Filename	LX-nm-2R-MSMS.d
ACQ Method	LX-NM-2-MSMS.m	Comment		Acquired Time	6/5/2020 9:44:27 PM (UTC+08:00)

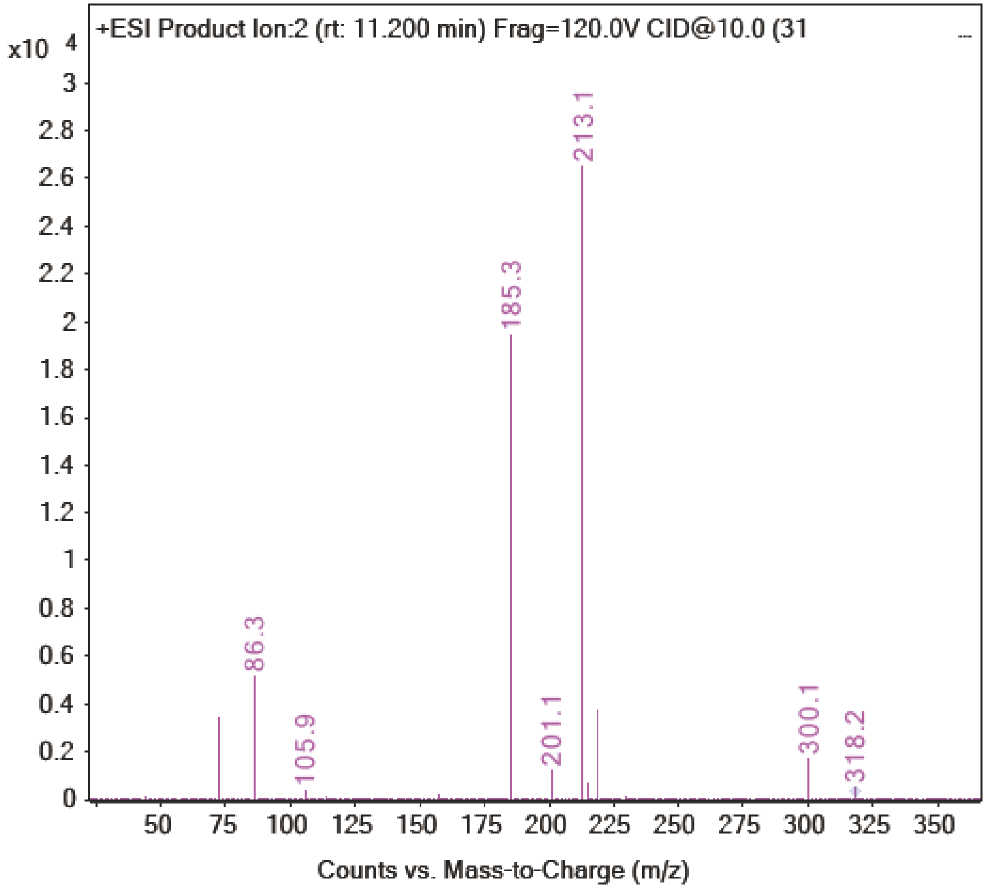

6. S6 MS/MS spectrum of V L(I)

Sample Name	LX-nm-2R-MSMS	Position	P1-A3	Instrument Name	Instrument 1
User Name	YU JIA	Inj Vol	10	InjPosition	
Sample Type	Sample	IRM Calibration Status	Not Applicable	Data Filename	LX-nm-2R-MSMS.d
ACQ Method	LX-NM-2-MSMS.m	Comment		Acquired Time	6/5/2020 9:44:27 PM (UTC+08:00)

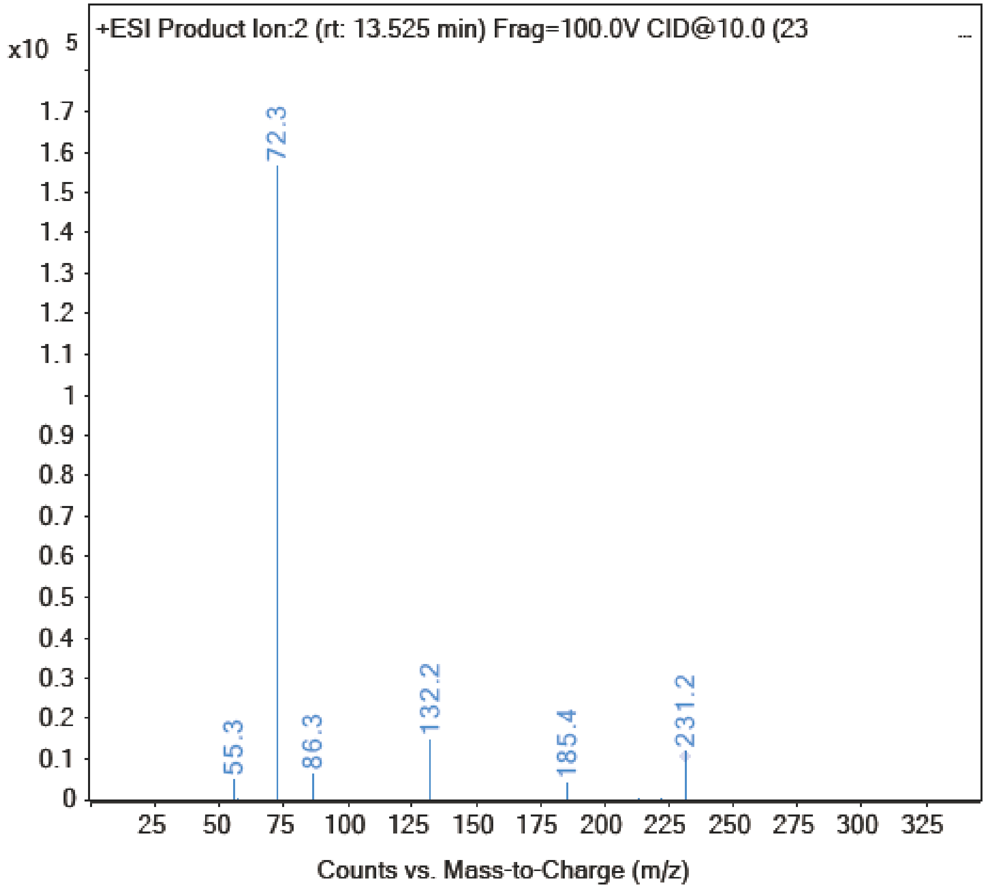

7. S7 MS/MS spectrum of FD

**Sample Name**	LX-nm-2R-MSMS	**Position**	P1-A3	**Instrument Name**	Instrument 1
**User Name**	YU JIA	**Inj Vol**	10	**InjPosition**	
**Sample Type**	Sample	**IRM Calibration Status**	Not Applicable	**Data Filename**	LX-nm-2R-MSMS.d
**ACQ Method**	LX-NM-2-MSMS.m	**Comment**		**Acquired Time**	6/5/2020 9:44:27 PM (UTC+08:00)

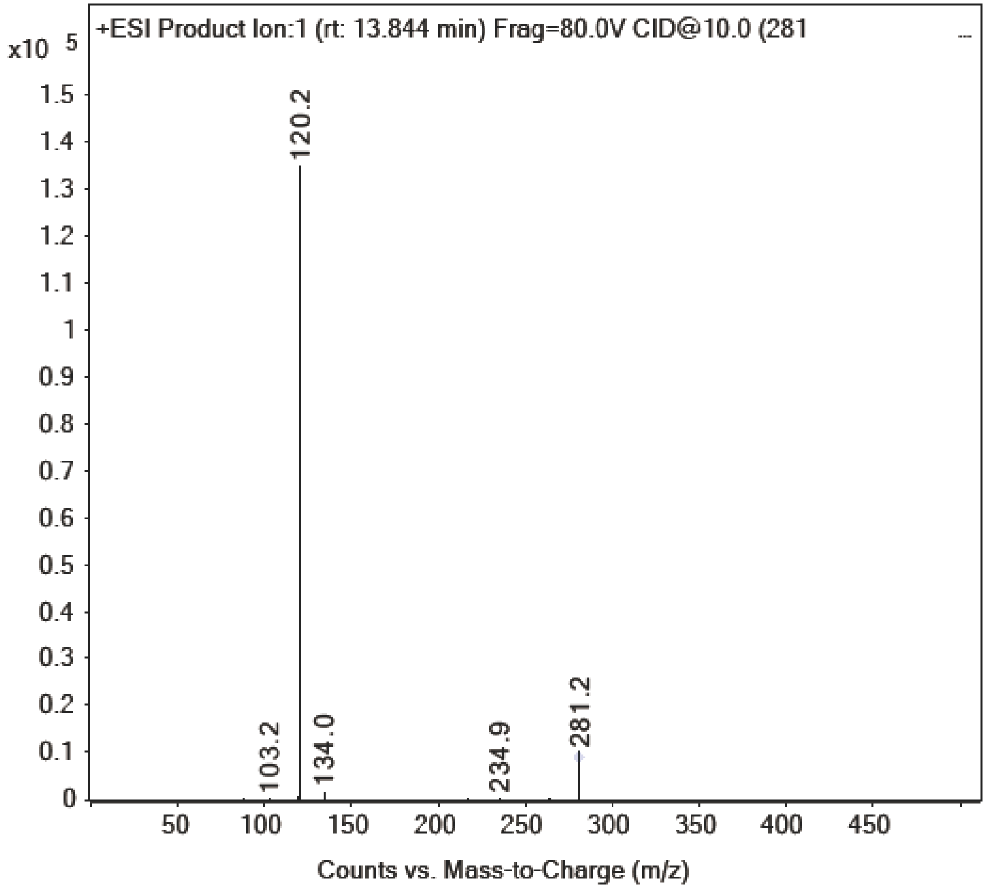

8. S8 MS/MS spectrum of L(I)L(I)

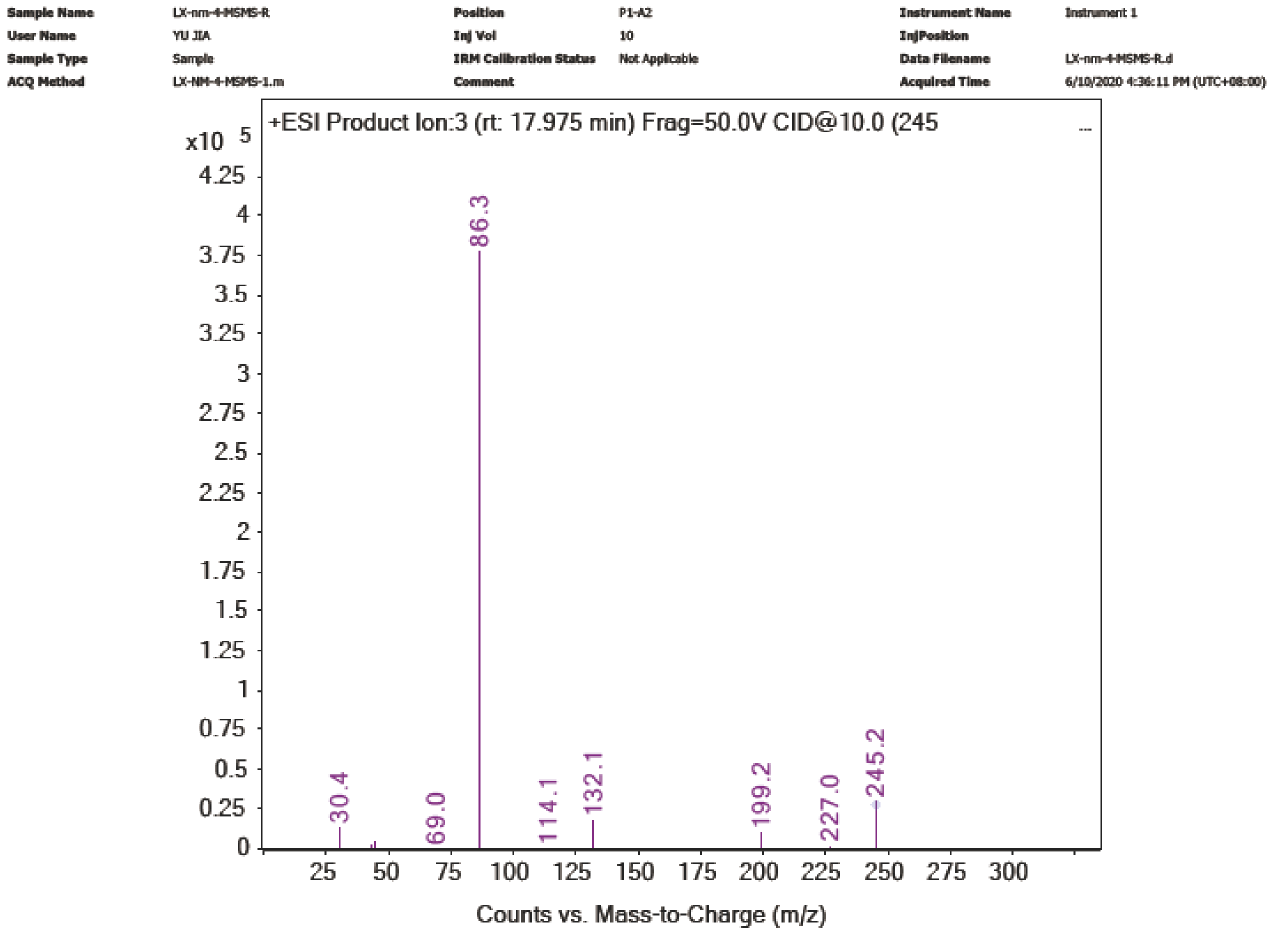

9. S9 MS/MS spectrum of DQ

**Sample Name**	LX-nm-4R-MSMS	**Position**	P1-A4	**Instrument Name**	Instrument 1
**User Name**	YU JIA	**Inj Vol**	10	**InjPosition**	
**Sample Type**	Sample	**IRM Calibration Status**	Not Applicable	**Data Filename**	LX-nm-4R-MSMS.d
**ACQ Method**	LX-NM-4-MSMS.m	**Comment**		**Acquired Time**	6/5/2020 10:32:30 PM (UTC+08:00)

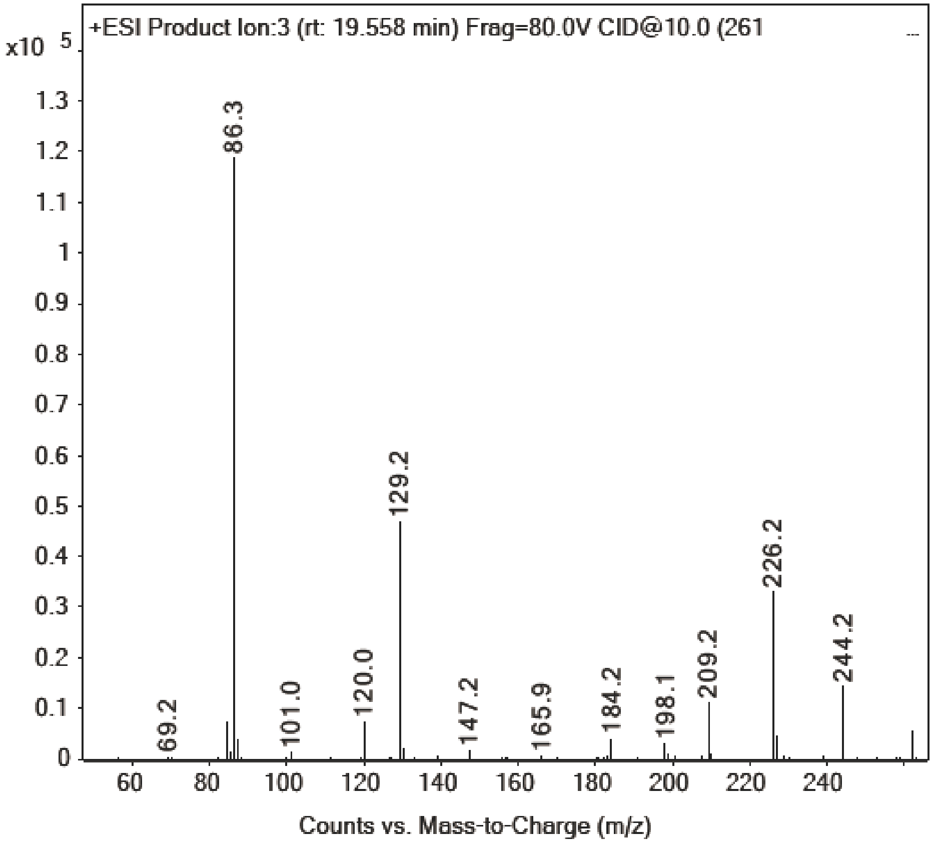

10. S10 MS/MS spectrum of VVYP

Sample Name	LX-nm-4R-MSMS	Position	P1-A4	Instrument Name	Instrument 1
User Name	YU JIA	Inj Vol	10	InjPosition	
Sample Type	Sample	IRM Calibration Status	Not Applicable	Data Filename	LX-nm-4R-MSMS.d
ACQ Method	LX-NM-4-MSMS.m	Comment		Acquired Time	6/5/2020 10:32:30 PM (UTC+08:00)

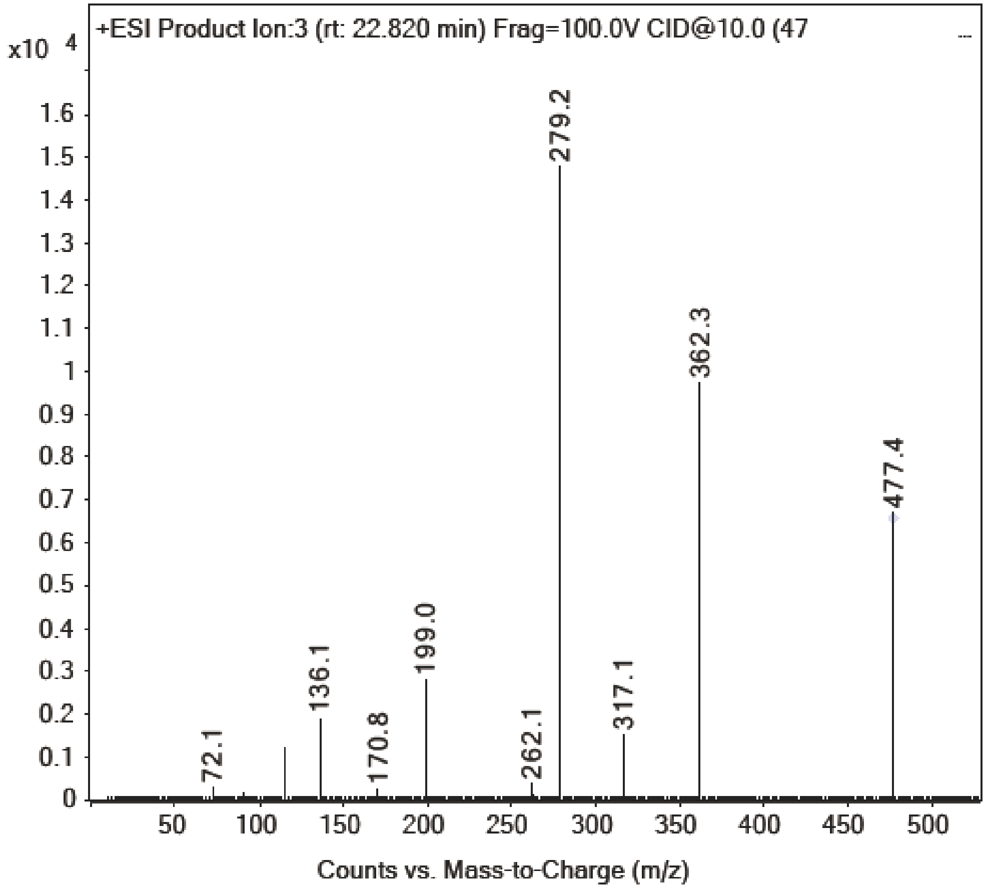

11. S11 MS/MS spectrum of FL(I)

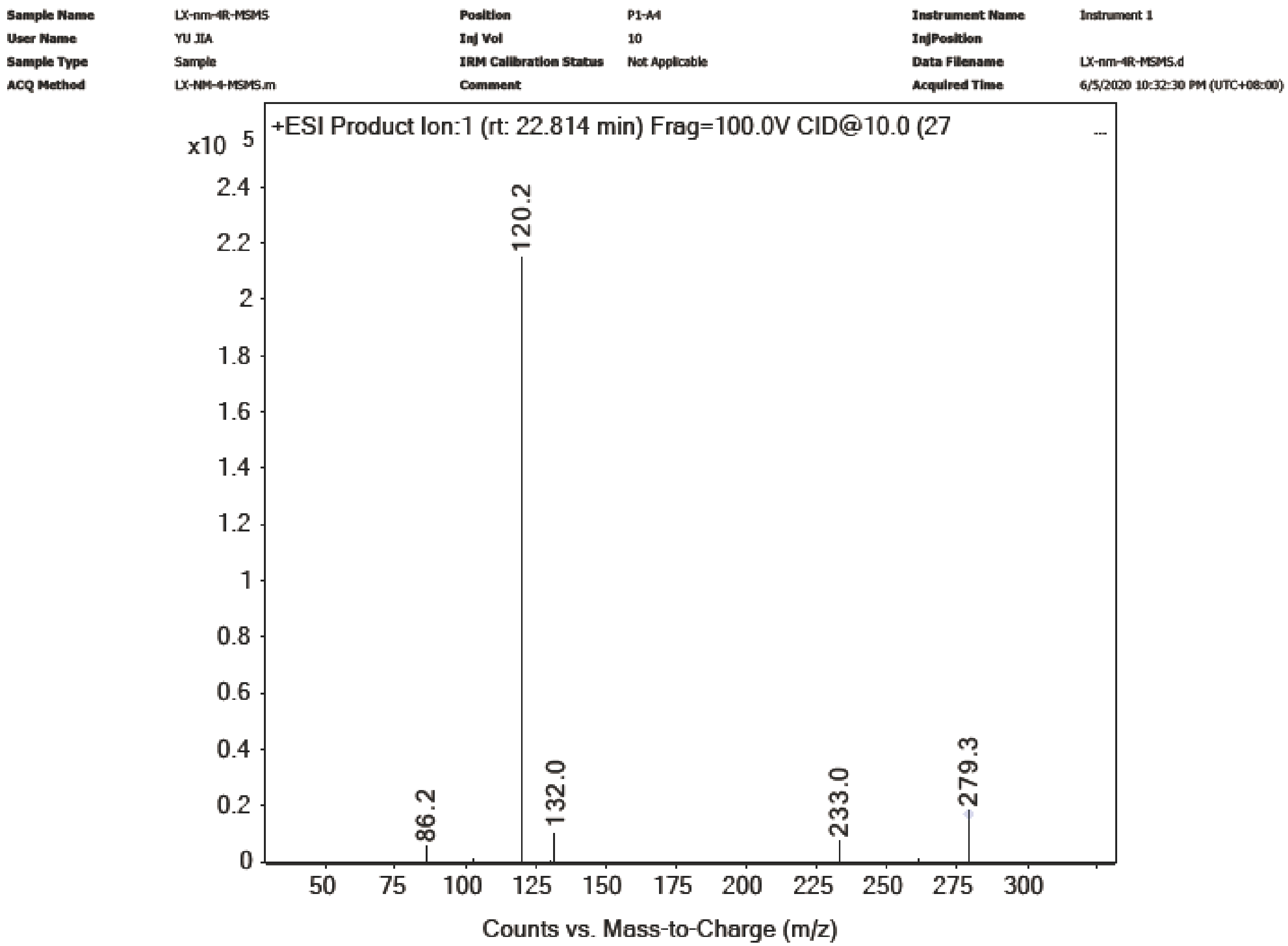

12. S12 MS/MS spectrum of VAN

**Sample Name**	LX-nm-1-MSMS-R	**Position**	P1-A1	**Instrument Name**	Instrument 1
**User Name**	YU JIA	**Inj Vol**	10	**InjPosition**	
**Sample Type**	Sample	**IRM Calibration Status**	Not Applicable	**Data Filename**	LX-nm-1MSMS-R.d
**ACQ Method**	LX-NM-1-MSMS.m	**Comment**		**Acquired Time**	6/9/2020 5:59:51 PM (UTC+08:00)

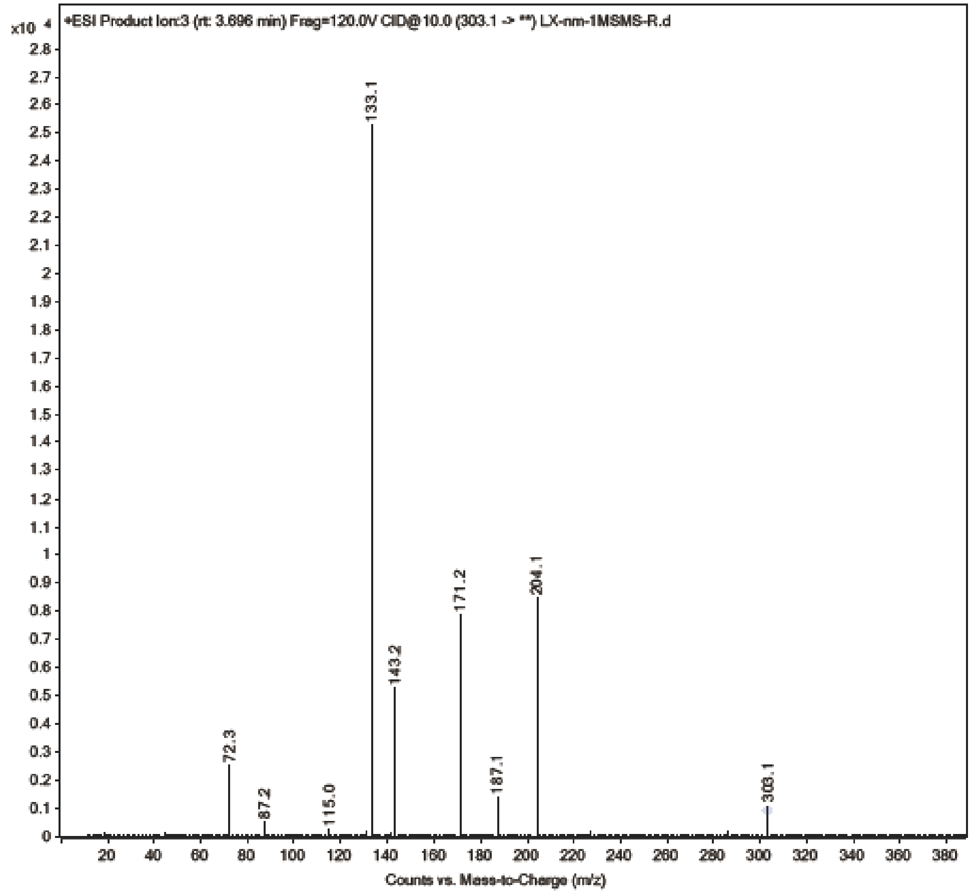

13. S13 MS/MS spectrum of L(I)L(I)VT

**Sample Name**	LX-nm-4R-MSMS	**Position**	P1-A4	**Instrument Name**	Instrument 1
**User Name**	YU JIA	**Inj Vol**	10	**InjPosition**	
**Sample Type**	Sample	**IRM Calibration Status**	Not Applicable	**Data Filename**	LX-nm-4R-MSMS.d
**ACQ Method**	LX-NM-4-MSMS.m	**Comment**		**Acquired Time**	6/5/2020 10:32:30 PM (UTC+08:00)

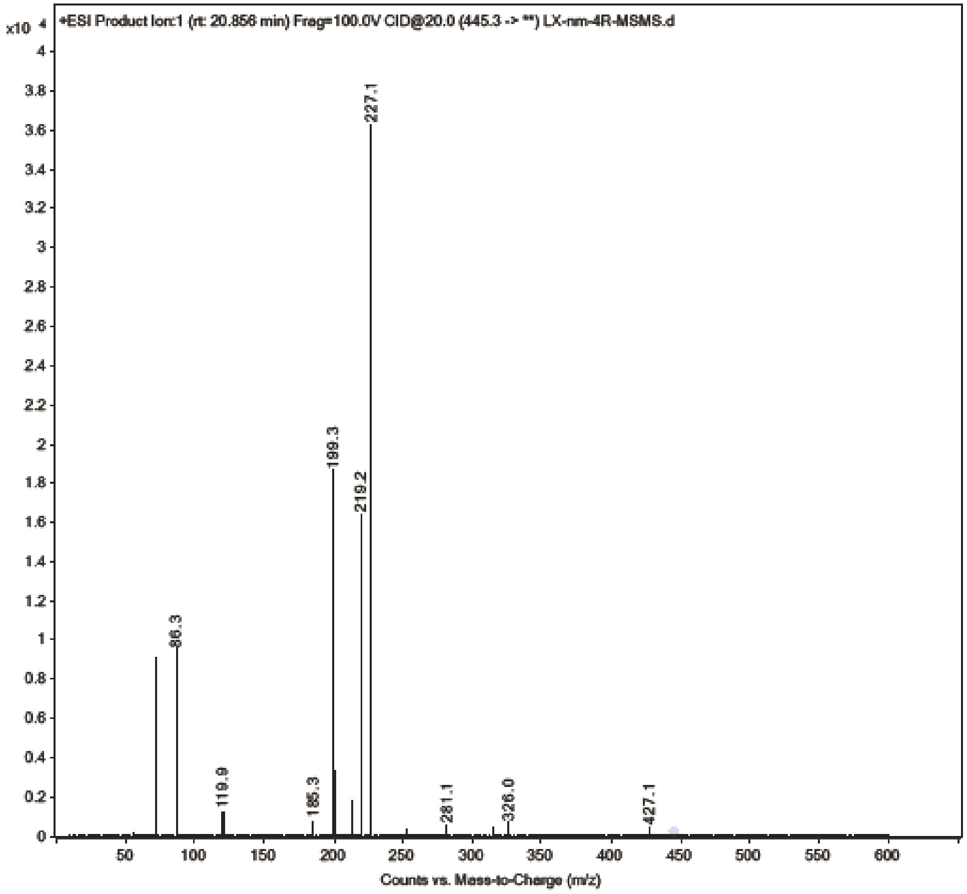

14. S14 MS/MS spectrum of PHPTT

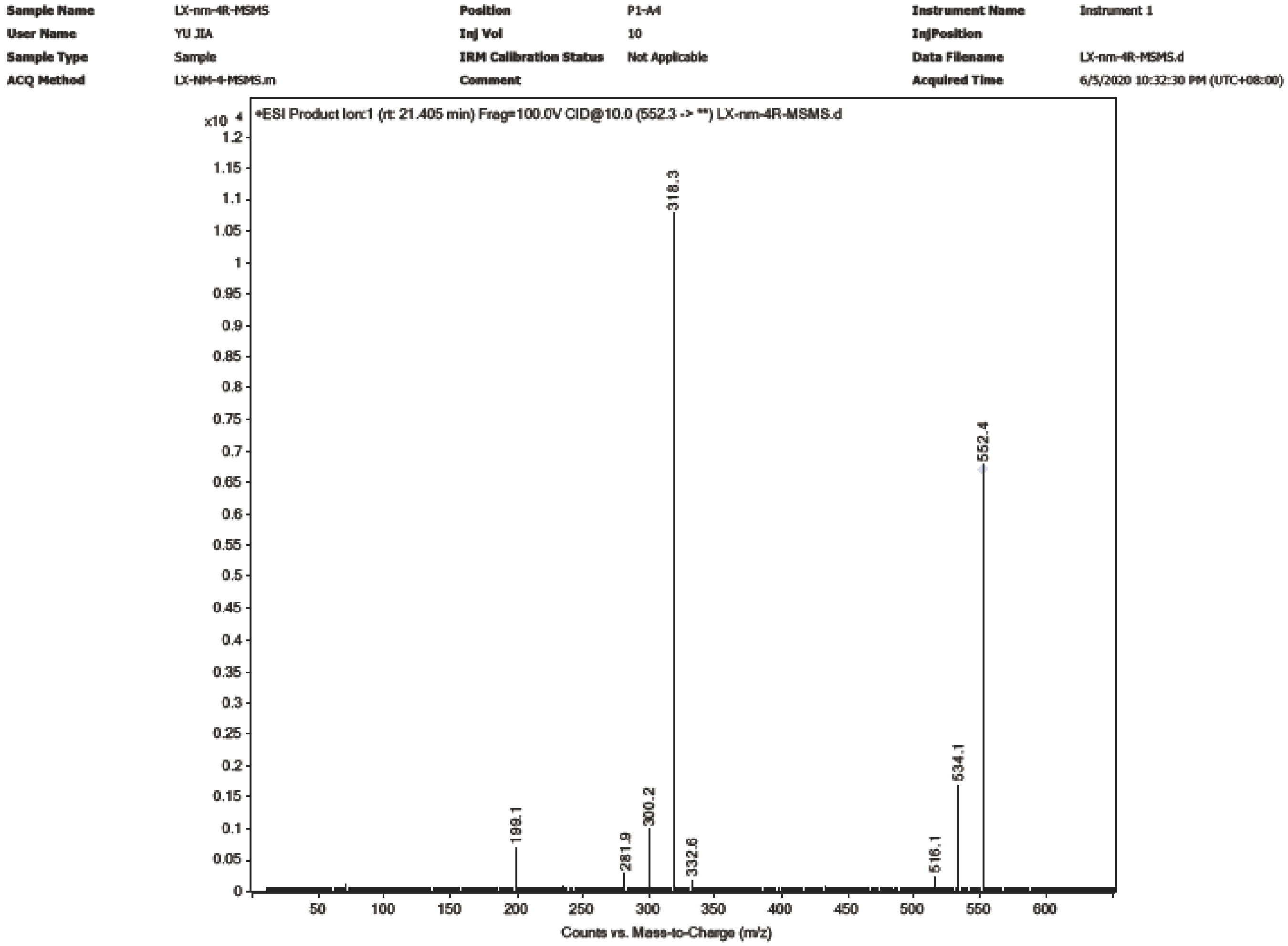

15. S15 MS/MS spectrum of EVAF

**Sample Name**	LX-nm-4R-MSMS	**Position**	P1-A4	**Instrument Name**	Instrument 1
**User Name**	YU JIA	**Inj Vol**	10	**InjPosition**	
**Sample Type**	Sample	**IRM Calibration Status**	Not Applicable	**Data Filename**	LX-nm-4R-MSMS.d
**ACQ Method**	LX-NM-4-MSMS.m	**Comment**		**Acquired Time**	6/5/2020 10:32:30 PM (UTC+08:00)

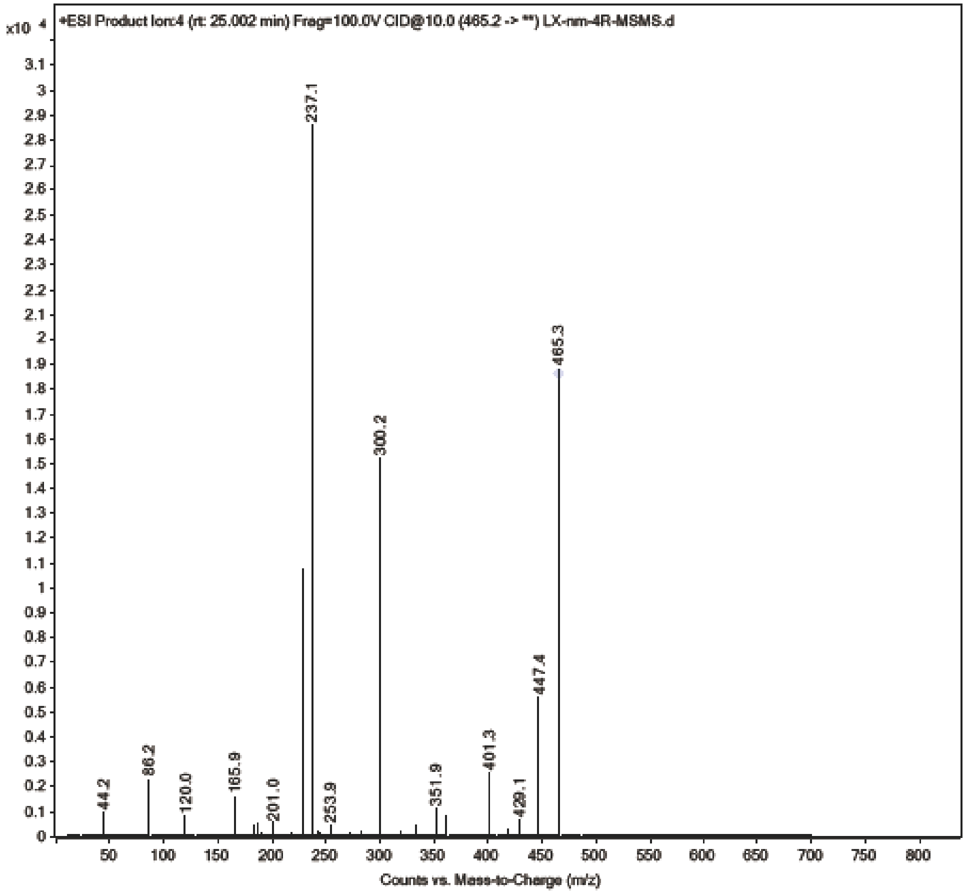

16. S16 MS/MS spectrum of VL(I)DAF

**Sample Name**	LX-nm-4R-MSMS	**Position**	P1-A4	**Instrument Name**	Instrument 1
**User Name**	YU JIA	**Inj Vol**	10	**InjPosition**	
**Sample Type**	Sample	**IRM Calibration Status**	Not Applicable	**Data Filename**	LX-nm-4R-MSMS.d
**ACQ Method**	LX-NM-4-MSMS.m	**Comment**		**Acquired Time**	6/5/2020 10:32:30 PM (UTC+08:00)

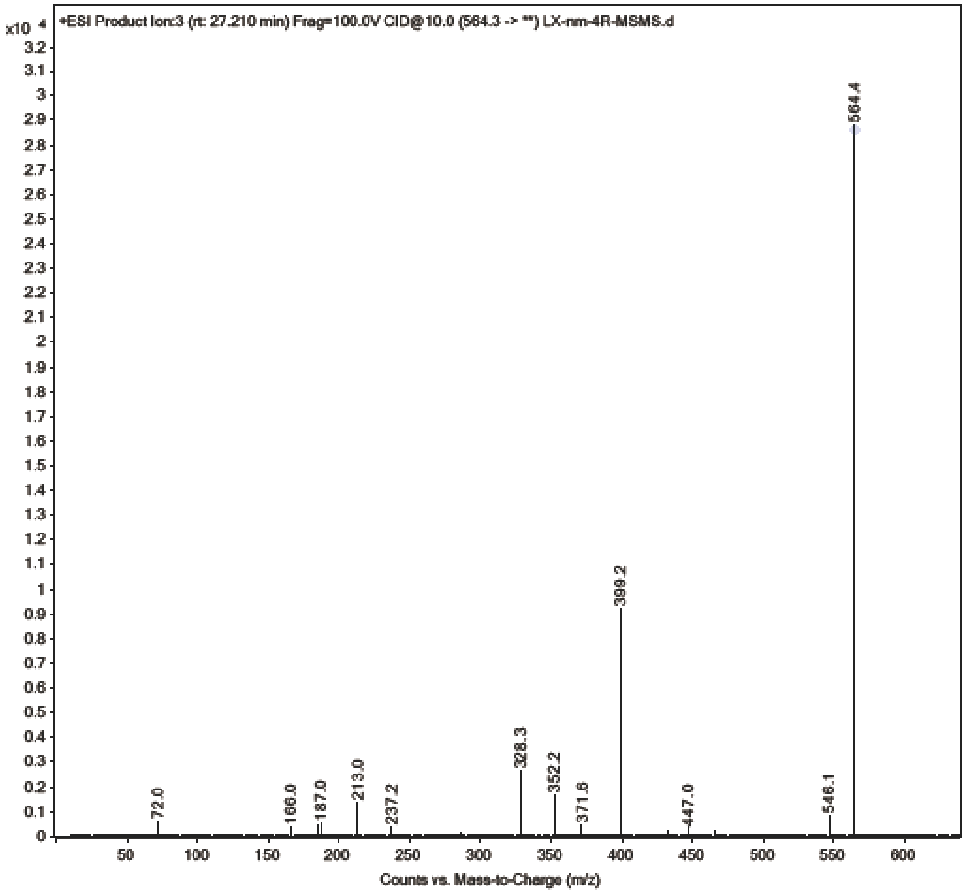

17. S17 MS/MS spectrum of L(I)H

Sample Name	LX-nm-1-MSMS-R	Position	P1-A1	Instrument Name	Instrument 1
User Name	YU JIA	Inj Vol	10	InjPosition	
Sample Type	Sample	IRM Calibration Status	Not Applicable	Data Filename	LX-nm-1MSMS-R.d
ACQ Method	LX-NM-1-MSMS.m	Comment		Acquired Time	6/9/2020 5:59:51 PM (UTC+08:00)

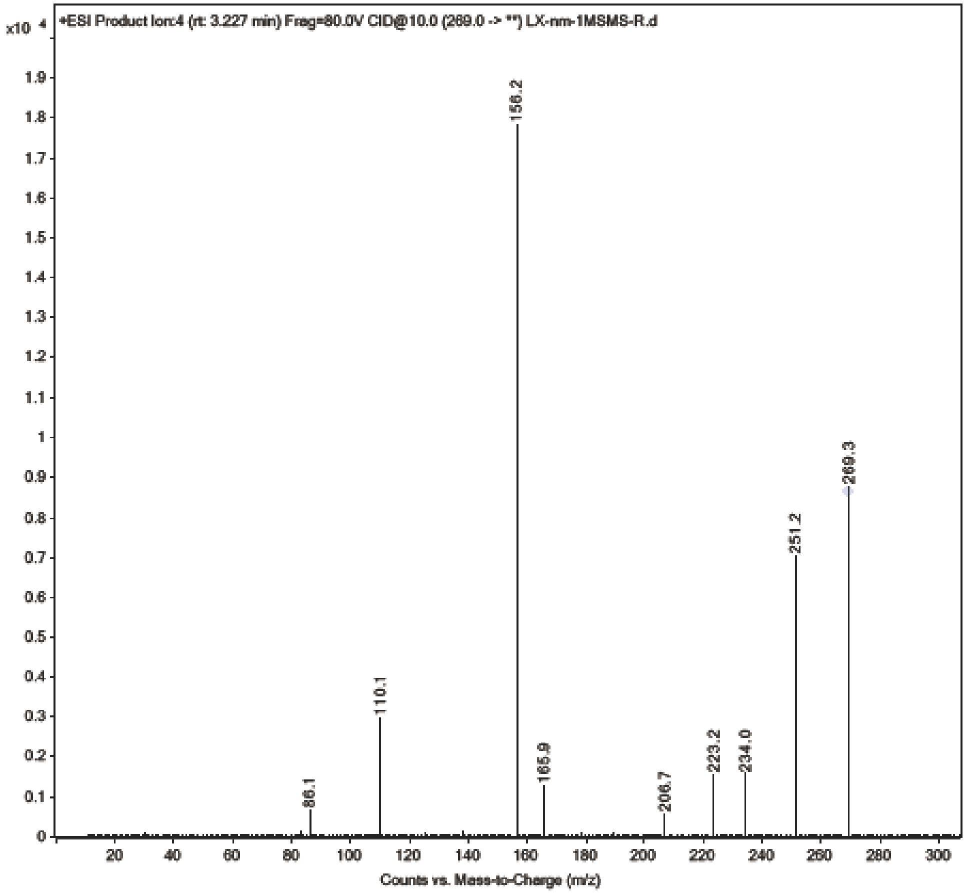

18. S18 MS/MS spectrum of VN

**Sample Name**	LX-nm-1-MSMS-R	**Position**	P1-A1	**Instrument Name**	Instrument 1
**User Name**	YU JIA	**Inj Vol**	10	**InjPosition**	
**Sample Type**	Sample	**IRM Calibration Status**	Not Applicable	**Data Filename**	LX-nm-1MSMS-R.d
**ACQ Method**	LX-NM-1-MSMS.m	**Comment**		**Acquired Time**	6/9/2020 5:59:51 PM (UTC+08:00)

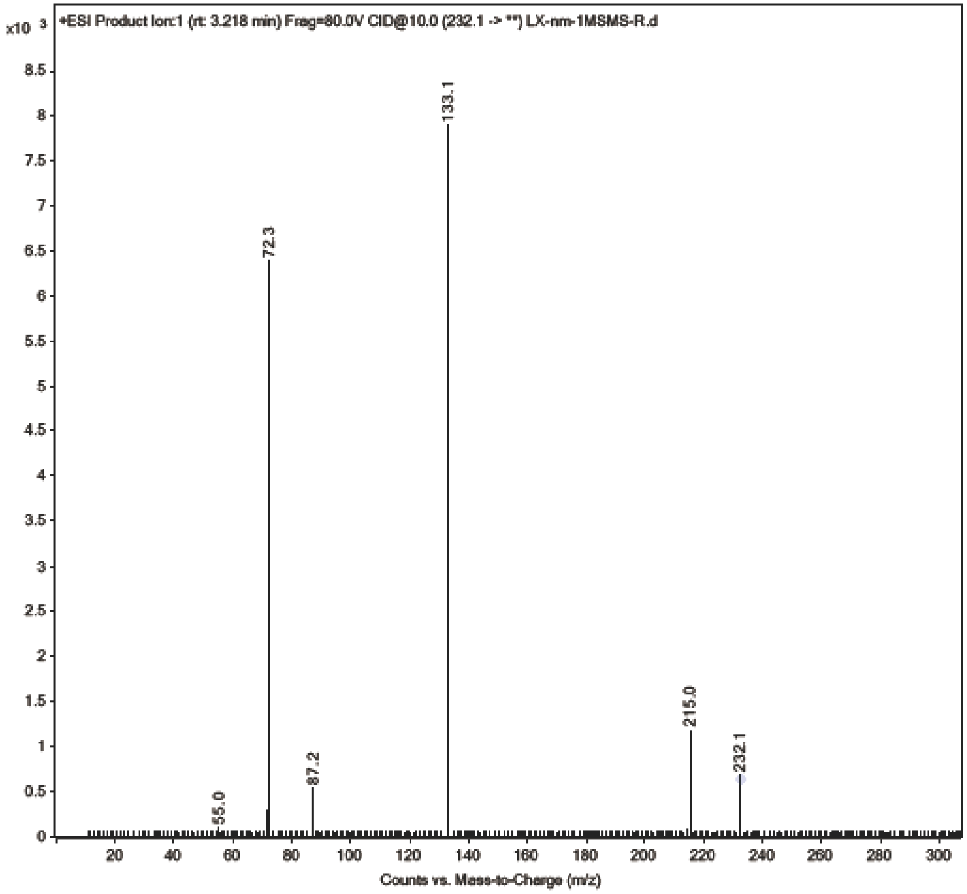

19. S19 MS/MS spectrum of VGH

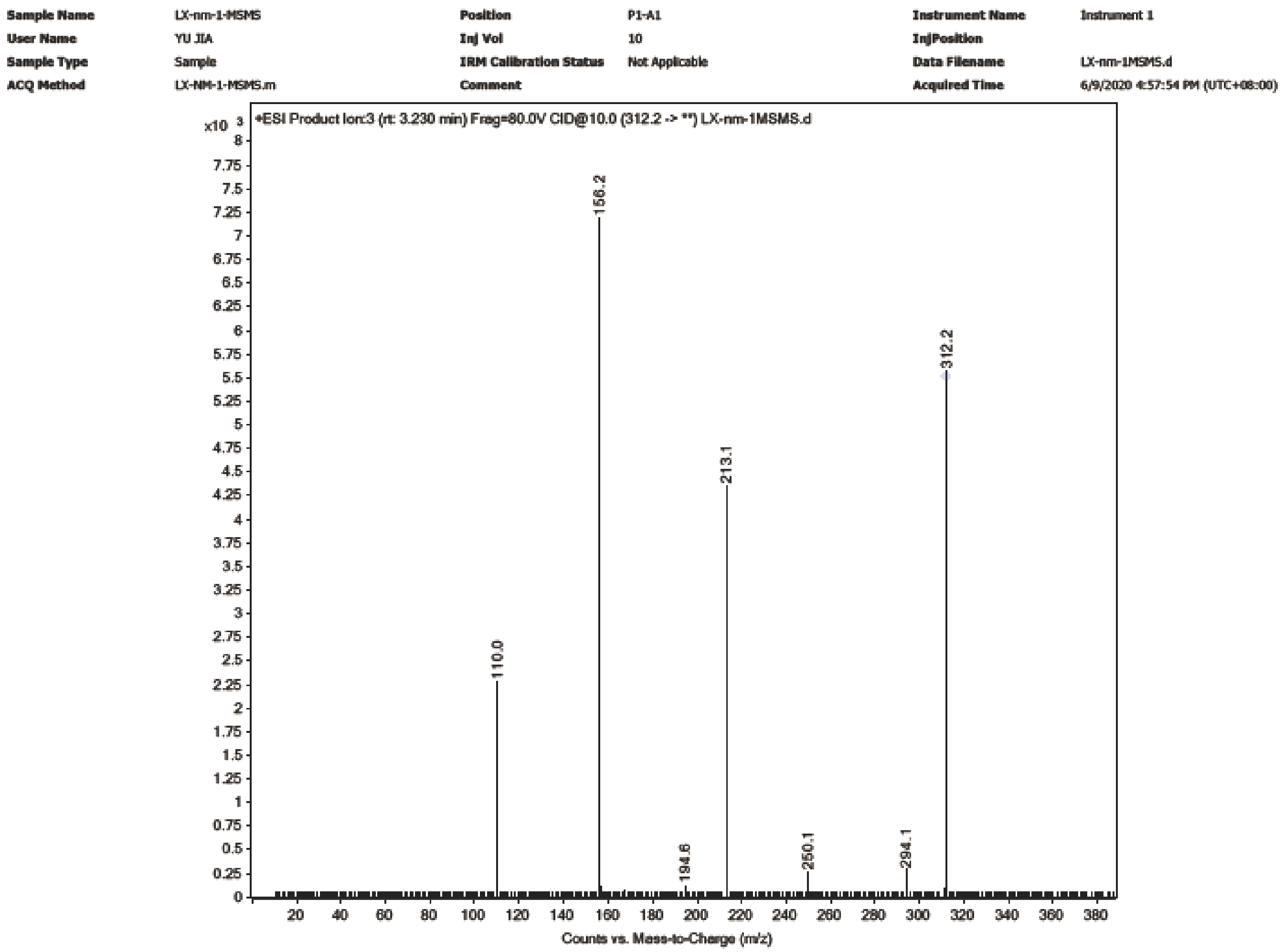

20. S20 MS/MS spectrum of VT

**Sample Name**	LX-nm-1-MSMS-R	**Position**	P1-A1	**Instrument Name**	Instrument 1
**User Name**	YU JIA	**Inj Vol**	10	**InjPosition**	
**Sample Type**	Sample	**IRM Calibration Status**	Not Applicable	**Data Filename**	LX-nm-1MSMS-R.d
**ACQ Method**	LX-NM-1-MSMS.m	**Comment**		**Acquired Time**	6/9/2020 5:59:51 PM (UTC+08:00)

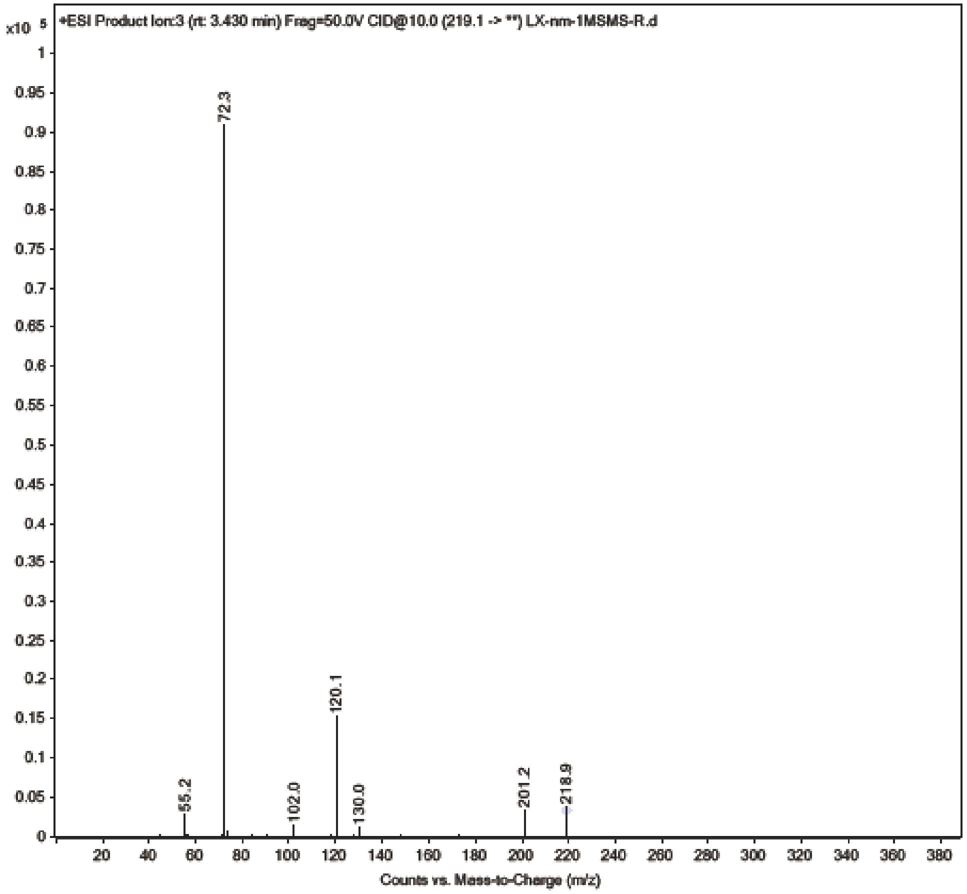

21. S21 MS/MS spectrum of TVA

Sample Name	LX-nm-1-MSMS-R	Position	P1-A1	Instrument Name	Instrument 1
User Name	YU JIA	Inj Vol	10	InjPosition	
Sample Type	Sample	IRM Calibration Status	Not Applicable	Data Filename	LX-nm-1MSMS-R.d
ACQ Method	LX-NM-1-MSMS.m	Comment		Acquired Time	6/9/2020 5:59:51 PM (UTC+08:00)

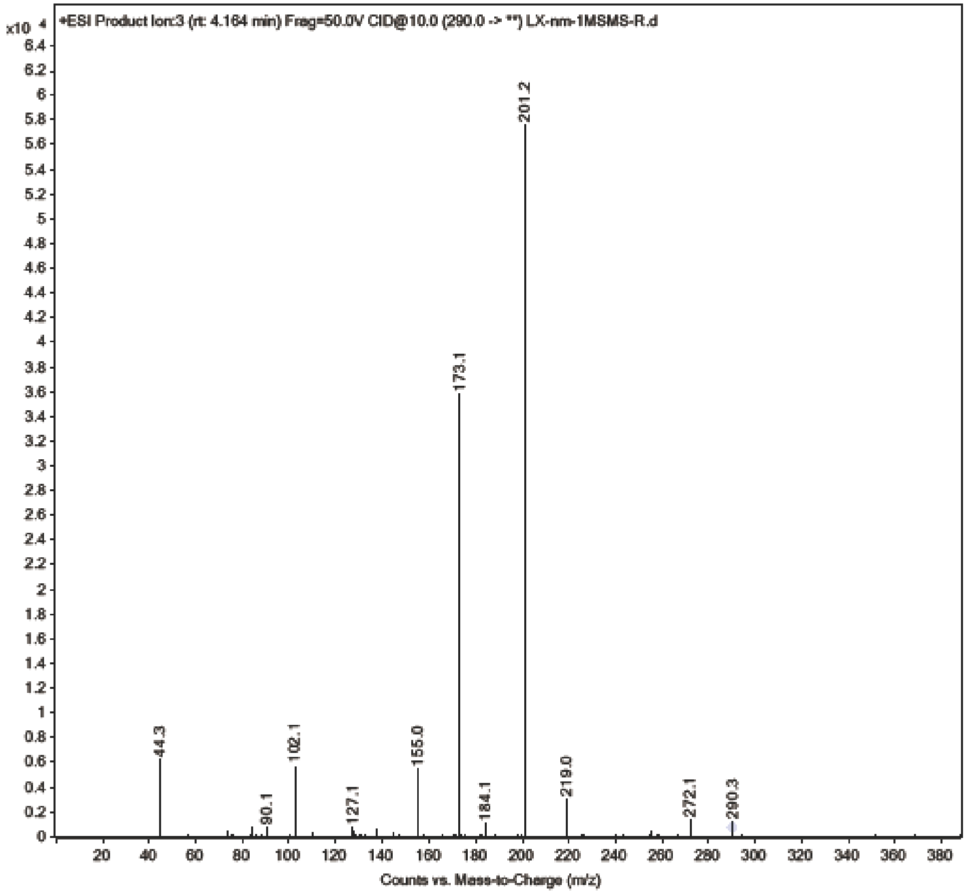

22. S22 MS/MS spectrum of L(I)Q

**Sample Name**	LX-nm-1-MSMS-R	**Position**	P1-A1	**Instrument Name**	Instrument 1
**User Name**	YU JIA	**Inj Vol**	10	**InjPosition**	
**Sample Type**	Sample	**IRM Calibration Status**	Not Applicable	**Data Filename**	LX-nm-1MSMS-R.d
**ACQ Method**	LX-NM-1-MSMS.m	**Comment**		**Acquired Time**	6/9/2020 5:59:51 PM (UTC+08:00)

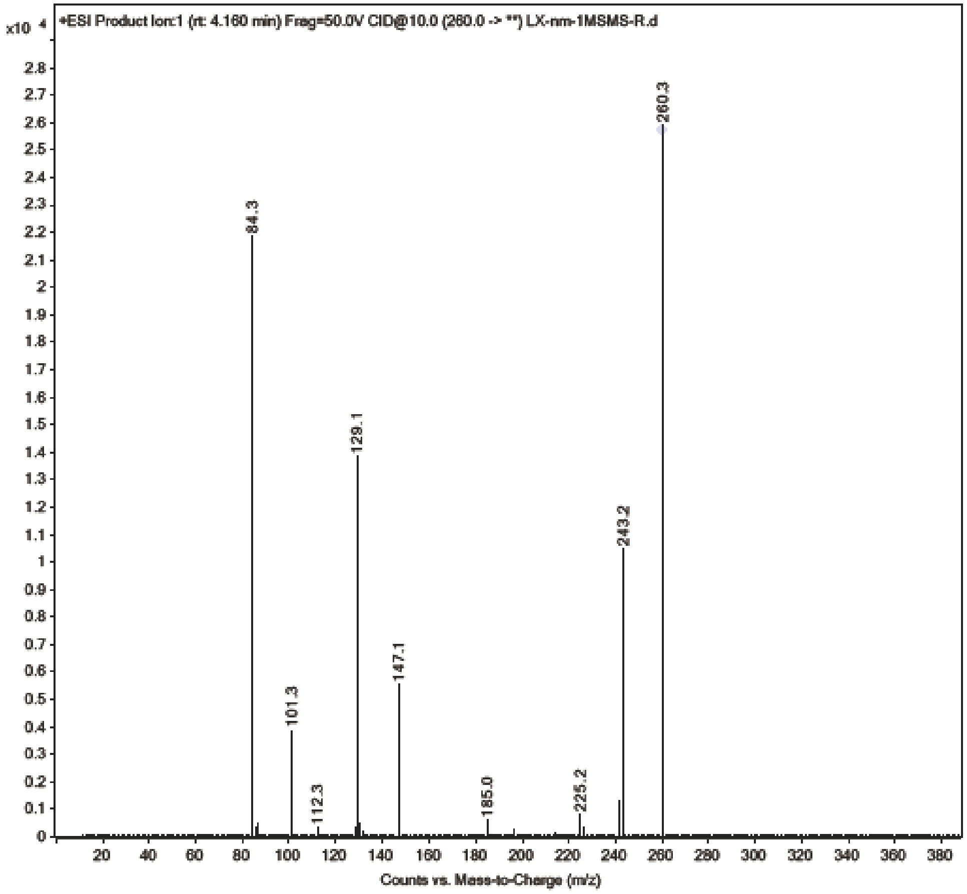

23. S23 MS/MS spectrum of L(I)

**Sample Name**	LX-nm-1-MSMS-R	**Position**	P1-A1	**Instrument Name**	Instrument 1
**User Name**	YU JIA	**Inj Vol**	10	**InjPosition**	
**Sample Type**	Sample	**IRM Calibration Status**	Not Applicable	**Data Filename**	LX-nm-1MSMS-R.d
**ACQ Method**	LX-NM-1-MSMS.m	**Comment**		**Acquired Time**	6/9/2020 5:59:51 PM (UTC+08:00)

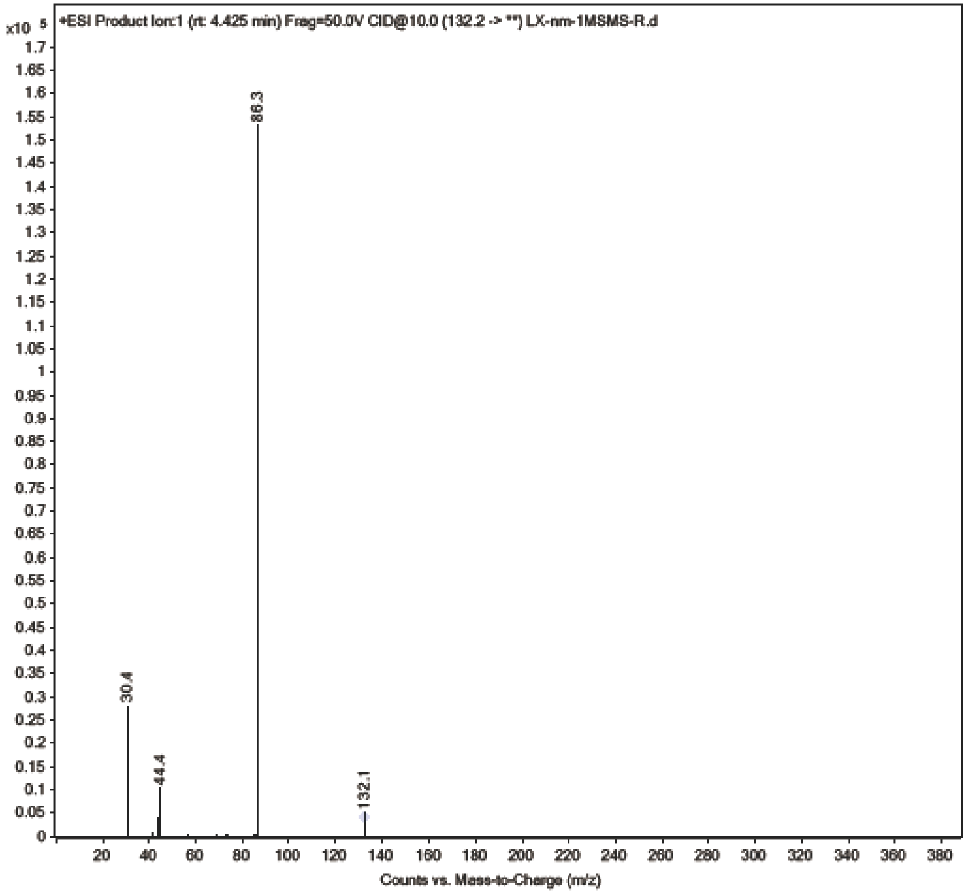

24. S24 MS/MS spectrum of L(I)T

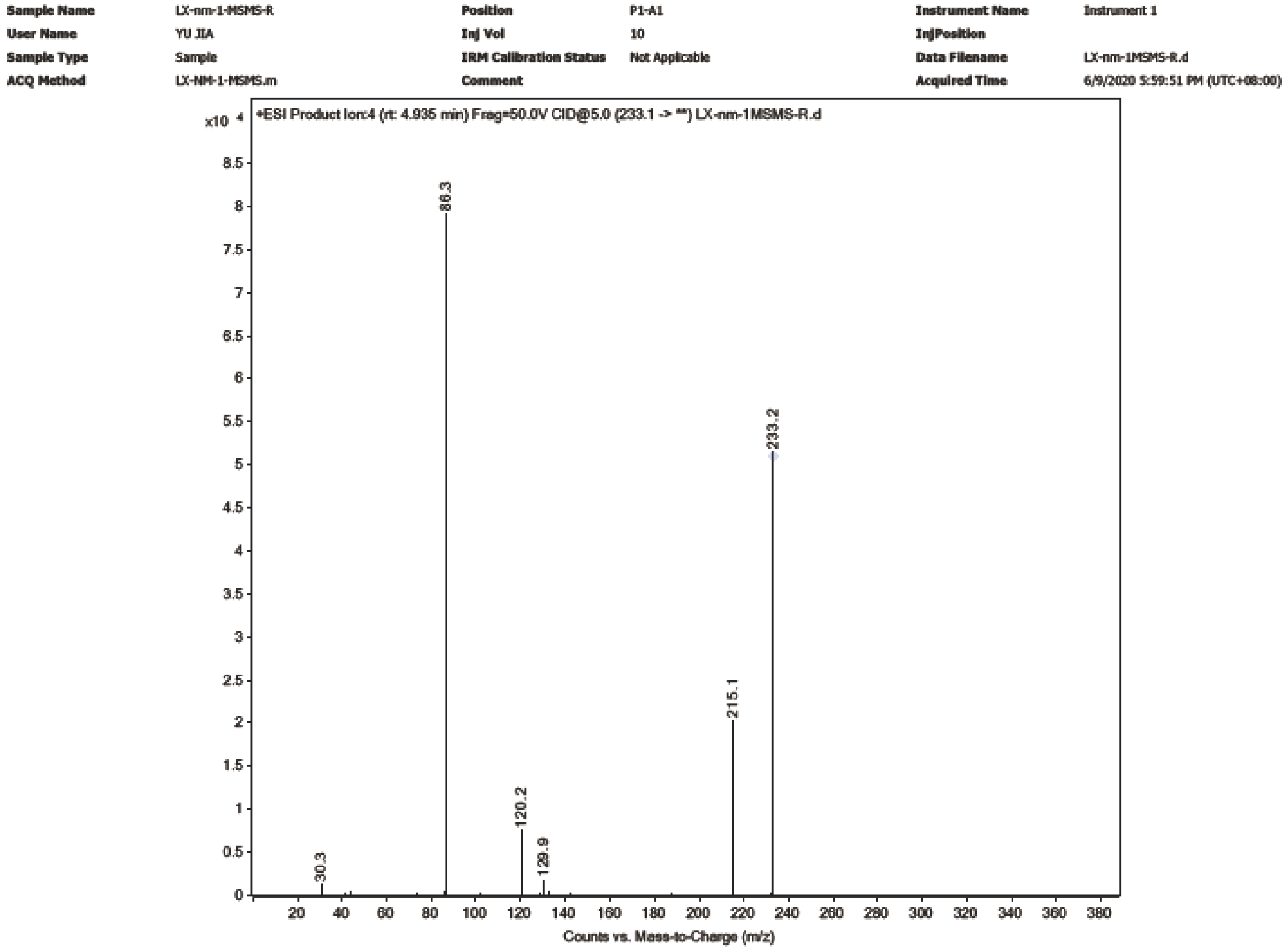

25. S25 MS/MS spectrum of AL(I)

**Sample Name**	LX-nm-1-MSMS-R	**Position**	P1-A1	**Instrument Name**	Instrument 1
**User Name**	YU JIA	**Inj Vol**	10	**InjPosition**	
**Sample Type**	Sample	**IRM Calibration Status**	Not Applicable	**Data Filename**	LX-nm-1MSMS-R.d
**ACQ Method**	LX-NM-1-MSMS.m	**Comment**		**Acquired Time**	6/9/2020 5:59:51 PM (UTC+08:00)

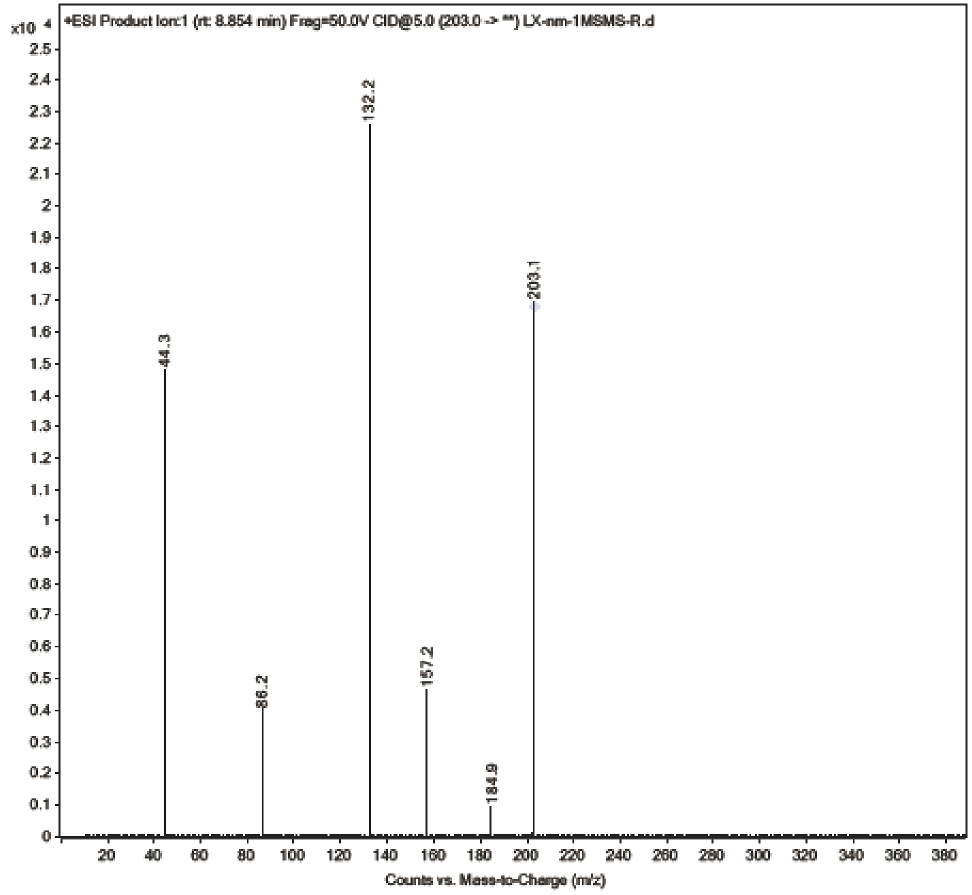

26. S26 MS/MS spectrum of L(I)SE

**Sample Name**	LX-nm-1-MSMS-R	**Position**	P1-A1	**Instrument Name**	Instrument 1
**User Name**	YU JIA	**Inj Vol**	10	**InjPosition**	
**Sample Type**	Sample	**IRM Calibration Status**	Not Applicable	**Data Filename**	LX-nm-1MSMS-R.d
**ACQ Method**	LX-NM-1-MSMS.m	**Comment**		**Acquired Time**	6/9/2020 5:59:51 PM (UTC+08:00)

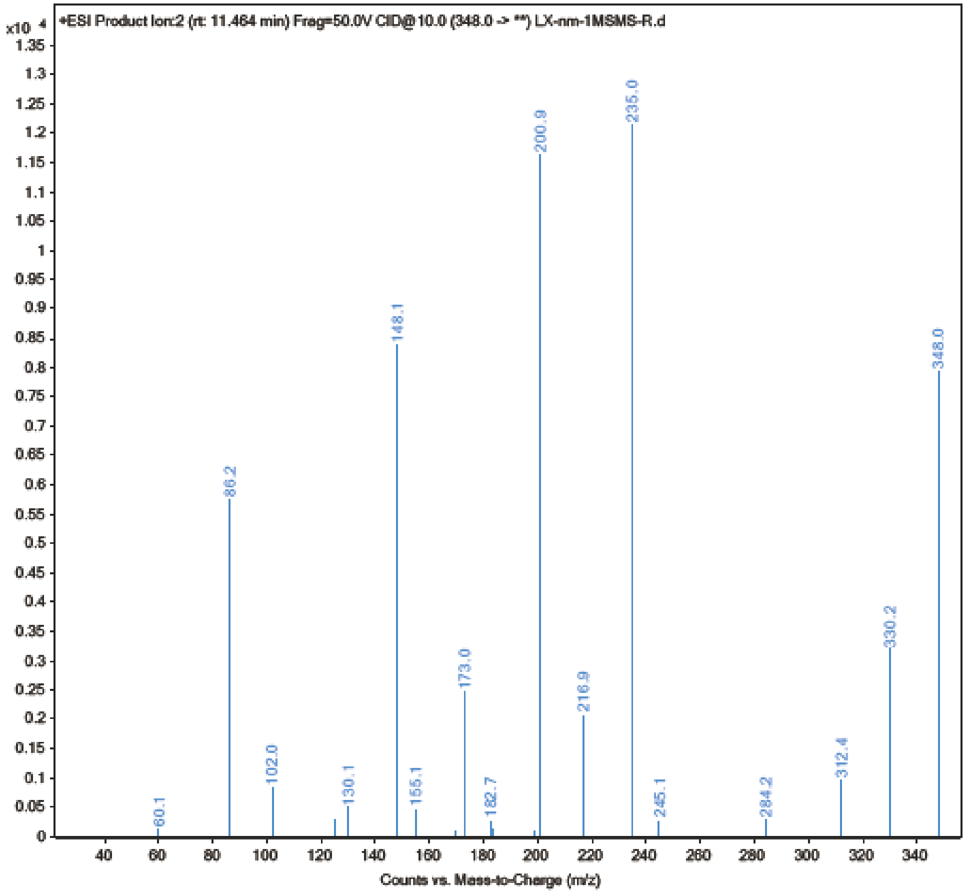

27. S27 MS/MS spectrum of VL(I)K

**Sample Name**	LX-nm-2R-MSMS	**Position**	P1-A3	**Instrument Name**	Instrument 1
**User Name**	YU JIA	**Inj Vol**	10	**InjPosition**	
**Sample Type**	Sample	**IRM Calibration Status**	Not Applicable	**Data Filename**	LX-nm-2R-MSMS.d
**ACQ Method**	LX-NM-2-MSMS.m	**Comment**		**Acquired Time**	6/5/2020 9:44:27 PM (UTC+08:00)

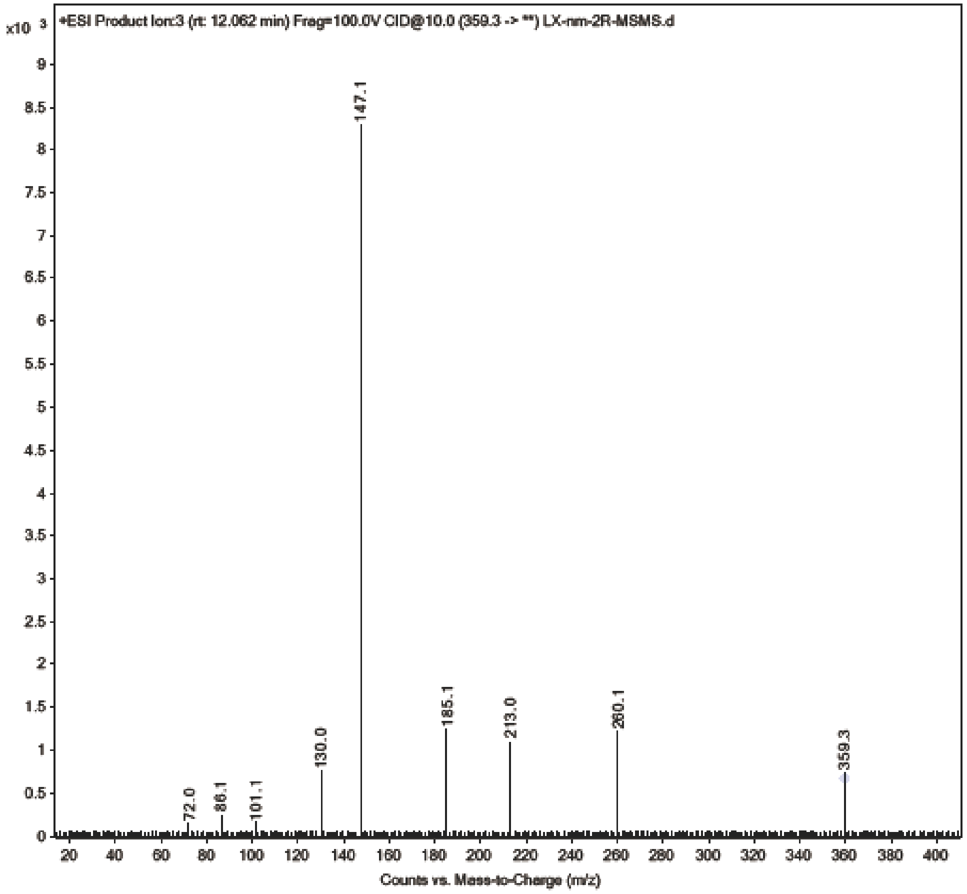

28. S28MS/MS spectrum of L(I)VT

**Sample Name**	LX-nm-2R-MSMS	**Position**	P1-A3	**Instrument Name**	Instrument 1
**User Name**	YU JIA	**Inj Vol**	10	**InjPosition**	
**Sample Type**	Sample	**IRM Calibration Status**	Not Applicable	**Data Filename**	LX-nm-2R-MSMS.d
**ACQ Method**	LX-NM-2-MSMS.m	**Comment**		**Acquired Time**	6/5/2020 9:44:27 PM (UTC+08:00)

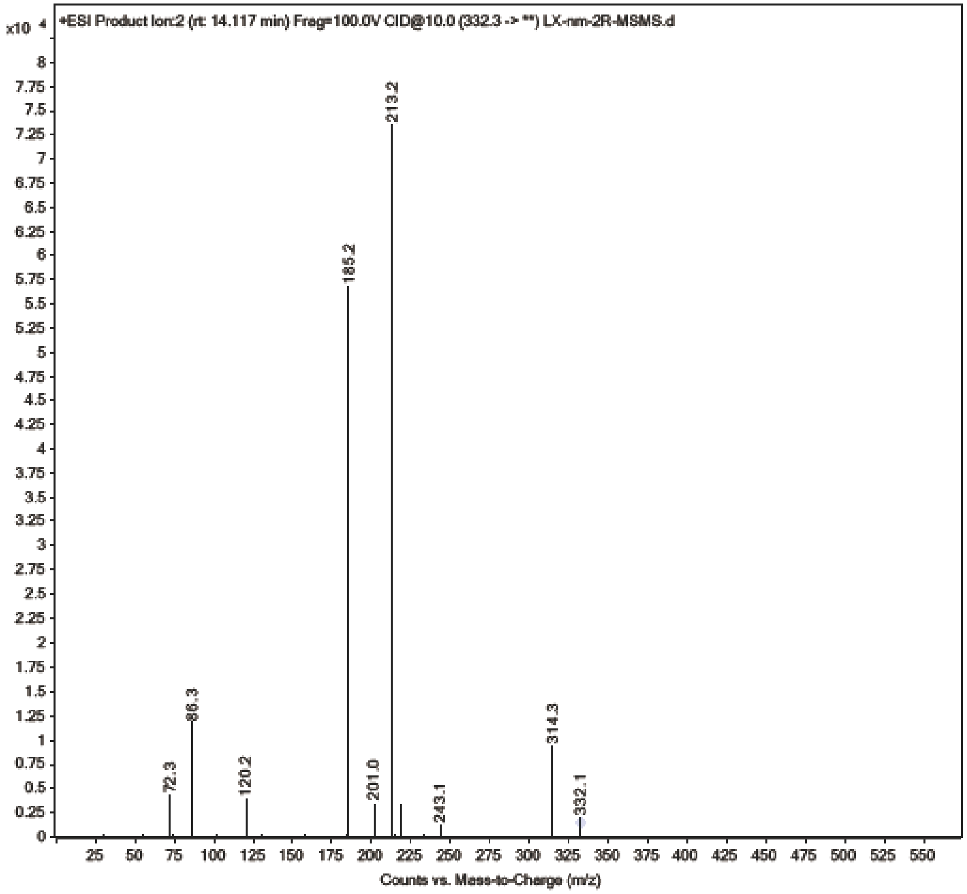

29. S29 MS/MS spectrum of L(I)VE

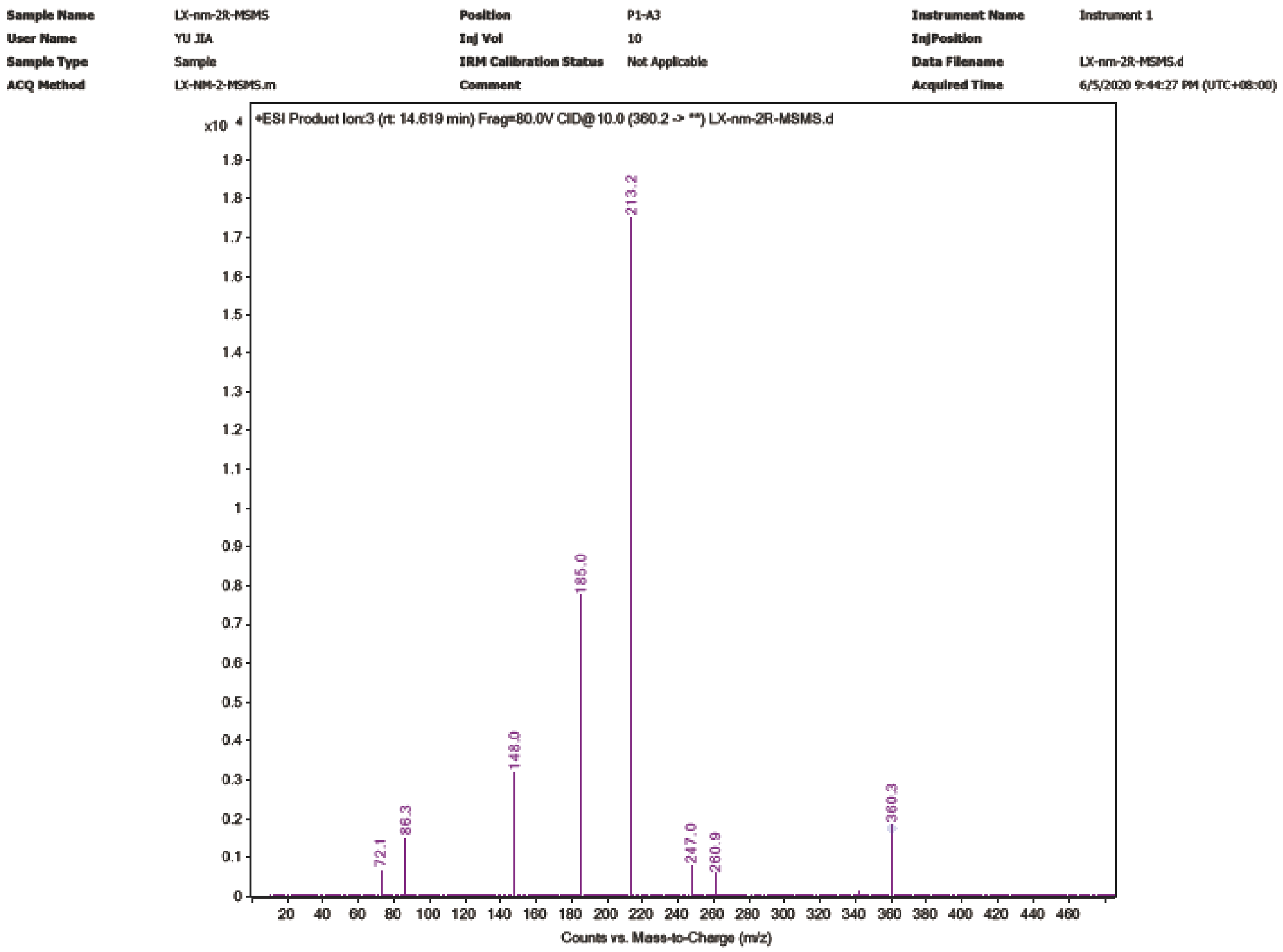

30. S30MS/MS spectrum of L(I)SAL(I)

**Sample Name**	LX-nm-2R-MSMS	**Position**	P1-A3	**Instrument Name**	Instrument 1
**User Name**	YU JIA	**Inj Vol**	10	**InjPosition**	
**Sample Type**	Sample	**IRM Calibration Status**	Not Applicable	**Data Filename**	LX-nm-2R-MSMS.d
**ACQ Method**	LX-NM-2-MSMS.m	**Comment**		**Acquired Time**	6/5/2020 9:44:27 PM (UTC+08:00)

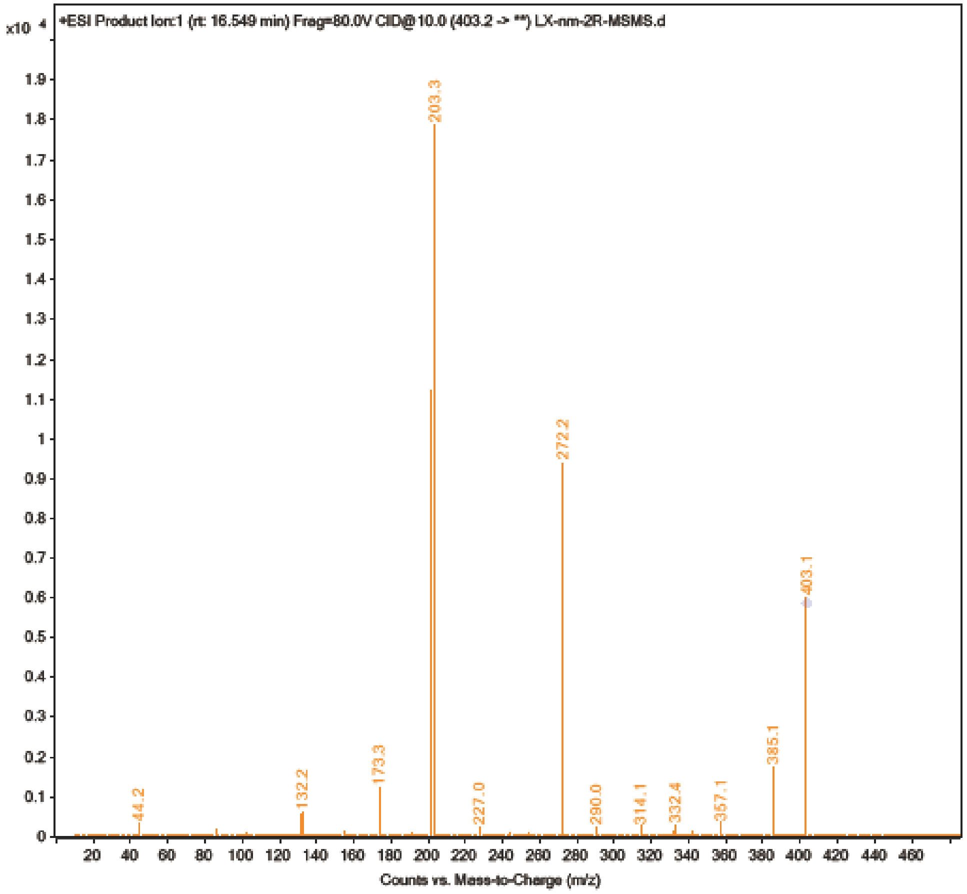

31. S31 MS/MS spectrum of AF

**Sample Name**	LX-nm-2R-MSMS	**Position**	P1-A3	**Instrument Name**	Instrument 1
**User Name**	YU JIA	**Inj Vol**	10	**InjPosition**	
**Sample Type**	Sample	**IRM Calibration Status**	Not Applicable	**Data Filename**	LX-nm-2R-MSMS.d
**ACQ Method**	LX-NM-2-MSMS.m	**Comment**		**Acquired Time**	6/5/2020 9:44:27 PM (UTC+08:00)

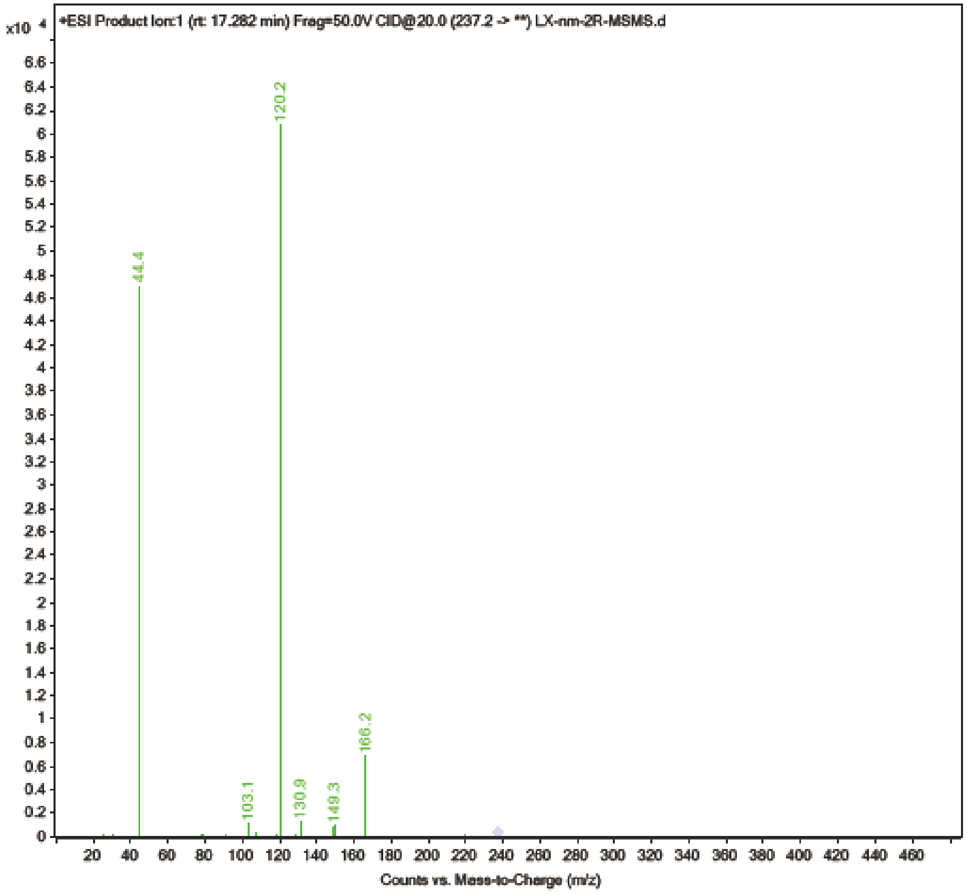

32. S32 MS/MS spectrum of WT

**Sample Name**	LX-nm-4R-MSMS	**Position**	P1-A4	**Instrument Name**	Instrument 1
**User Name**	YU JIA	**Inj Vol**	10	**InjPosition**	
**Sample Type**	Sample	**IRM Calibration Status**	Not Applicable	**Data Filename**	LX-nm-4R-MSMS.d
**ACQ Method**	LX-NM-4-MSMS.m	**Comment**		**Acquired Time**	6/5/2020 10:32:30 PM (UTC+08:00)

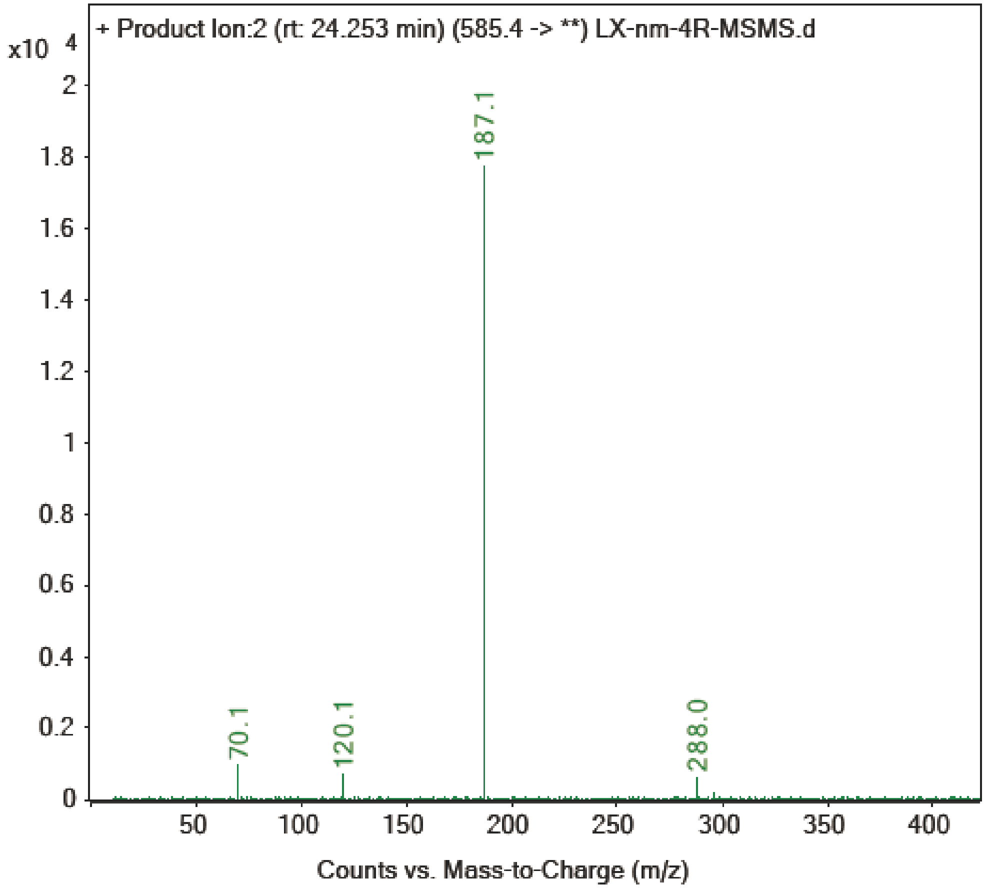

Supplement: Supplementary file 1 [file molecules-26-07614-s001.zip › molecules-1477652-supplementary.pdf]
